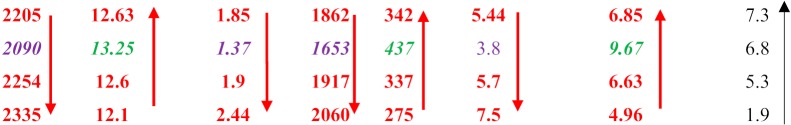# On the Microstructure and Isothermal Oxidation at 800 and 1200 °C of the Nb-24Ti-18Si-5Al-5Cr-5Ge-5Sn (at.%) Silicide-Based Alloy

**DOI:** 10.3390/ma13030722

**Published:** 2020-02-05

**Authors:** Ofelia Hernández-Negrete, Panos Tsakiropoulos

**Affiliations:** Department of Materials Science and Engineering, Sir Robert Hadfield Building, The University of Sheffield, Mappin Street, Sheffield S1 3JD, UK; ochernandeznegrete@gmail.com

**Keywords:** Nb-silicide-based alloys, high-entropy alloys, complex concentrated alloys, pest oxidation, high temperature oxidation, intermetallics, silicides

## Abstract

The research presented in this paper aspired to understand how the simultaneous addition of Ge and Sn in an Hf-free Nb-silicide-based alloy affected its oxidation resistance. Results are presented for the Nb-24Ti-18Si-5Al-5Cr-5Ge-5Sn alloy (at.%) which was studied in the as-cast and heat-treated (1400 °C/100 h) conditions and after isothermal oxidation in air at 800 and 1200 °C. There was macrosegregation in the cast alloy, in which the Nb_ss_ formed at a low volume fraction and was not stable after heat treatment at 1400 °C. The βNb_5_Si_3_, A15-Nb_3_Sn, and C14-NbCr_2_ were stable phases. The alloy did not undergo pest oxidation at 800 °C, and there was no spallation of its scale at 1200 °C. There was enrichment in Ge and Sn in the substrate below the scale/substrate interface, where the compounds Nb_3_Sn, Nb_5_Sn_2_Si, (Ti,Nb)_6_Sn_5_, and Nb_5_Ge_3_ were formed. After the oxidation at 1200 °C, the solid solution in the bulk of the alloy was very Ti-rich (Ti,Nb)_ss_. Improvement of oxidation resistance at both temperatures was accompanied by a decrease and increase, respectively, of the alloy parameters VEC (valence electron concentration) and δ, in agreement with the alloy design methodology NICE (Niobium Intermetallic Composite Elaboration). The elimination of scale spallation at 1200 °C was attributed (a) to the formation of Ti-rich (Ti,Nb)_ss_ solid solution and (Ti,Nb)_6_Sn_5_, respectively, in the bulk and below the scale, (b) to the low concentration of Cr in the scale, (c) to the absence of GeO_2_ in the scale, (d) to the formation of αAl_2_O_3_ in the scale, and (e) to the presence (i) of Nb_5_Ge_3_ below the scale/substrate interface and (ii) of oxides in the scale, namely, SiO_2_, Al_2_O_3_, TiO_2_, and SnO_2_, and Ti_2_Nb_10_O_29_,TiNb_2_O_7_, and AlNbO_4_, respectively, with a range of intrinsic thermal shock resistances and coefficient of thermal expansion (CTE) values that reduced stresses in the scale and the substrate below it.

## 1. Introduction

Refractory metal intermetallic composites (RMICs) are considered to be potential replacements for Ni-based superalloys in future aero-engines owing to their higher solidus temperatures compared with Ni, and their promise of meeting property goals [1,2,3]. Nb-silicide-based alloys (also known as Nb-silicide in situ composites, Nb-silicide-based composites, or Nb in situ composites) can be cast and can offer low densities with outstanding balance of mechanical properties at temperatures between 1000 and 1400 °C coupled with oxidation resistance [1,4,5,6,7]. The latter was improved significantly to meet industry requirements for oxidation behavior at intermediate and high temperatures [2,3,8]. These new materials could be considered for practical applications provided they offer an optimum balance of mechanical and oxidation properties.

In multicomponent Nb-silicide-based alloys, the most common phases are alloyed Nb_ss_ (cI2-W), Nb_3_Si (tP32-Ti_3_P), αNb_5_Si_3_ (tI32-Cr_5_B_3_), βNb_5_Si_3_ (tI32-W_5_Si_3_), and C14-NbCr_2_ (hP12-MgZn_2_) [2,9,10]. In the alloys that were studied to date, as many as 18 elements (Al, B, Ce, Cr, Fe, Ga, Ge, Hf, Ho, Mo, Si, Sn, Ta, Ti, V, W, Y, Zr) were used, albeit not all 18 in the same alloy. Some of these additions are believed to be essential for specific roles, i.e., some for oxidation resistance, others for high temperature strength and creep, and others for fracture toughness while some are thought to be required for more than one of these properties. Alloy chemistry and processing history are important for the type, volume fraction, size, and spatial distribution of phases in the microstructure, as well as for their properties [10]. For example, the alloying of the Nb_ss_ can improve high-temperature oxidation resistance without compromising other high-temperature mechanical properties [11,12,13], and the alloying of Nb_5_Si_3_ and C14-NbCr_2_ Laves phase can change the hardness and creep properties of these compounds [9,14,15].

It is well known that specific alloying additions in Nb enhance its oxidation resistance [11,16,17]. The latter increases with additions of Al, B, Cr, Ge, Hf, Mo, Si, Sn, and Ti in Nb-silicide-based alloys [5,10,18,19,20,21,22,23,24,25,26], in which Si is the key addition needed to form silicide(s) and for oxidation. Silica can be stable to higher temperatures than alumina, has a lower activation energy for oxygen diffusion, and could be more effective than alumina at higher temperatures [27]. Amorphous silica has some fluidity to heal cracks in the scale. Alumina is crystalline with virtually no fluidity. The oxides of the alloying elements Al, Hf, and Ti are even more thermodynamically stable than silica. The latter has virtually no solubility with Nb_2_O_5_ in the solid state. B_2_O_3_ or GeO_2_ in silica gives (a) a glass with lower viscosity at low temperatures where the resulting higher fluidity can improve the self-healing of the scale, and (b) a higher coefficient of thermal expansion (CTE) than pure silica [28]. The Nb-silicide-based alloys form complex oxide scales during oxidation [10,18,22,23,25,26,29]. The binary phase diagrams for these oxides with Nb_2_O_5_, which has a lower (about 1550 °C) melting point than Nb (2477 °C), indicate eutectic formation [30], which requires that the maximum useful application temperature should be lower that the eutectic temperatures.

The ability to cast Nb-silicide-based alloys is a great advantage over other RMICs [4]. However, owing to their very high liquidus temperatures, cold hearth processing is required, and macrosegregation can be severe in these materials depending on alloying additions [31]. Tin, Ge, or B have a strong effect on macrosegregation [32,33,34,35,36]. The additions of Sn or Ge, which can play a key role in the suppression of pest oxidation [2,20,21,22,32,33,37,38], can also reduce the alloy liquidus temperature, depending on their concentration and other alloying elements, for example, Al, Cr, Hf, Mo, and Ti. It is believed that their role in the alloy to achieve a balance of mechanical and oxidation properties depends on their concentration in the alloy. For example, it was suggested [2,37] that Sn can be effective at concentrations up to 2 at.% and that, at this “low” content, the A15-Nb_3_Sn compound is not stable. This was recently shown not to be the case [32].

Research on the oxidation of Nb-silicide-based alloys considered alloys where Sn or Ge was individually or simultaneously in synergy with Al, Cr, Si, and Ti, plus Hf and other transition and refractory metals. For example, Menon et al. [18] studied the alloys Nb-19.9Ti-19.7Si-4.2Ge-3.3Al-4.2Hf-9.9Cr, Nb-26Ti-12.6Si-4.9Ge-1.9Al-1.9Hf-6.7Cr-0.4Sn, and Nb-25.5Ti-14.9Si-4.5Ge-6.1Cr-1.6Hf-1.9Al-1.6Sn, Chan [19] studied the Nb-(21.5–26.6)Ti-(1–17.3)Si-(2.5–15.6)Cr-(2–4.7)Hf-(3–5.1)Ge alloys, Geng et al. [20] studied the alloy Nb-24Ti-18Si-5Al-5Cr-2Mo-5Hf-5Sn, Vellios [21] studied the alloys Nb-23Ti-5Si-5Al-5Hf-5V-2Cr-2Sn and Nb-30Ti-10Si-5Cr-5Sn-3Fe-2Al-2Hf, and Knittel et al. [22] studied the alloys Nb-25Ti-16Si-8Hf-2Al-2Cr-xSn (x = 0, 2, 4, 5, 6, 8) (all concentrations in at.%). More simple alloys were studied by Xu et al. [32,33] and Li and Tsakiropoulos [34,38], who sought to advance current understanding about how the synergy of Sn or Ge with Al and/or Cr improves the oxidation of Nb-24Ti-18Si-based alloys.

In Nb-silicide-based alloys the solubility of Sn in the solid solution is higher than in the Nb_5_Si_3_, and the opposite is the case for Ge [14,34,35]. In the Nb_5_Si_3_, the solubilities of Ge or Sn increase with the Ti concentration in the silicide [14]. Furthermore, the solubility of Si plus the elements that substitute it in Nb_5_Si_3_ decreases below the stoichiometric Si concentration in binary Nb_5_Si_3_ when Ge or Sn is in the solution as an individual addition [14]. The alloying of Nb_5_Si_3_ with Ge or Sn respectively increases and decreases the hardness compared with the binary silicide [14]. Tin in synergy with Al and Cr in Nb-24Ti-18Si-based alloys without Hf and other transition and refractory metals promotes the βNb_5_Si_3_→αNb_5_Si_3_ transformation [32,33]. The same is the case when Ge is in synergy with Al, Cr, and Hf without transition and refractory metals [34], but not the case without Hf and other transition and refractory metals [34]. The type of Nb_5_Si_3_ in the microstructure is important owing to the different properties and CTE of the binary and alloyed βNb_5_Si_3_ and αNb_5_Si_3_ silicides [14,39].

The studied concentration ranges of Sn and Ge additions in Nb-silicide-based alloys were wider for the former (2 to 8 at.%), compared with the latter [18,19,20,21,22,32,33,34,35]. The replacement of Si with 5 at.% Ge in the alloys studied by Menon et al. [18] appeared to have a remarkably favorable influence on the onset of breakaway oxidation, which was delayed by at least one order of magnitude in time. The addition of 5 at.% Ge in Nb-24Ti-18Si-5Cr-5Al-5Ge (alloy ZF6 in Reference [34]) suppressed the pest oxidation but not the oxide scale spallation at high temperature [38]. The same was the case in the Nb-24Ti-18Si-5Al-5Cr-2Sn (alloy ZX7 in Reference [32]) and Nb-24Ti-18Si-5Al-5Cr-5Sn (alloy ZX8 in Reference [33]. In other words, alloying with Sn suppresses pest oxidation at low (2 at.%) and higher concentrations but not the spallation of the scale at high temperatures [22,32,33]. The addition of Ge has the same effect as Sn [19,38]. Spallation of the scale occurred in the alloys that were studied by Menon et al. [18].

The Nb_ss_ is the Achilles’ heel in the oxidation of Nb-silicide-based alloys. The literature suggests that Sn can affect the stability of the Nb_ss_ in Nb-silicide-based alloys [33,35]. For example, alloying with Sn destabilized the Nb_ss_ in the alloy ZX6 (Nb-24Ti-18Si-5Al-5Sn) [33] and in Nb-18Si-5Al-5Sn (alloy EZ7 in Reference [35]) but Ge did not have a similar effect on the Nb_ss_ in Nb-24Ti-18Si-based alloys where Al or Cr was present individually or simultaneously or where Ge was in synergy with Al, Cr, and Hf in the alloy [34,40,41]. The Nb_ss_ was stable in the alloys Nb-23Ti-5Si-5Al-5Hf-5V-2Cr-2Sn and Nb-30Ti-10Si-5Cr-5Sn-3Fe-2Al-2Hf [21]. No solid solution was observed in the as-cast Nb-22.5Ti-17.3Si-15.6Cr-4Hf-4.8Ge, but the Nb_ss_ was stable (22 vol.%) after heat treatment (HT) at 1350 °C/100 h [19]. The Nb_ss_ was present in the cast alloys Nb-19.9Ti-19.7Si-4.2Ge-3.3Al-4.2Hf-9.9Cr and Nb-26Ti-12.6Si-4.9Ge-1.9Al-1.9Hf-6.7Cr-0.4Sn that were studied by Menon et al. [18].

The research on the oxidation of Nb-silicide-based alloys would suggest (a) that Ge and/or Sn are needed to suppress pest oxidation, and (b) that, when these alloying additions were present individually or simultaneously in the alloys, the spallation of their scales at high temperatures was not suppressed. It is now well established that Ge or Sn has an effect on macrosegregation and can also reduce the liquidus temperature of the alloy. Alloying with Sn can affect the stability of the Nb_ss_ but not the alloying with Ge, which can, however, significantly reduce the cracking of the substrate below the scale/substrate interface [38]. Unfortunately, from the oxidation research on multicomponent Nb-silicide-based alloys, it is not clear whether it was the synergy of Sn or Ge individually or simultaneously with Al, Cr, Si, and Ti that improved oxidation resistance, or whether the presence of oxygen scavenging Hf with/without transition and refractory metals and with some or all of the aforementioned elements was essential for increasing the oxidation resistance.

There is a significant cost difference for Ge and Sn; for example, the cost of the former is in the range $1550 to $1750 per kg compared with about $80 per kg for the latter. Do we need both these elements in Nb-silicide-based alloys to improve their oxidation resistance? What would be the effect of the simultaneous presence of Ge and Sn and the Ge/Sn ratio in Nb-silicide-based alloys on (i) macrosegregation, (ii) pest oxidation, (iii) scale spallation, (iv) Nb_ss_ stability, (v) the βNb_5_Si_3_ → αNb_5_Si_3_ transformation, and (vi) the microstructure of the substrate below the scale/substrate interface?

The motivation for the research presented in this paper was to answer these questions. We selected a model alloy based on the (reference) alloy Nb-24Ti-18Si-5Al-5Cr (alloy KZ5 in Reference [42]) with Ge/Sn = 1 and equal concentrations of Ge and Sn, each at 5 at.%, guided by the research reported in References [10,33,38], to assist us to understand how Ge and Sn “work together”. The nominal composition (at.%) of the alloy was Nb-24Ti-18Si-5Al-5Cr-5Ge-5Sn (alloy OHS1), and it was compared with the reference alloy KZ5 and the equivalent alloys with only Sn (Nb-14Ti-18Si-5Al-5Cr-5Sn, alloy ZX8 in Reference [33]) or Ge (Nb-24Ti-18Si-5Al-5Cr-5Ge, alloy ZF6 in Reference [34]) addition. The alloy was prepared using arc melting, the same as for the alloys KZ5, ZX8, and ZF6.

The structure of the paper is as follows: firstly, the microstructures of the cast and heat-treated alloy are discussed, followed by the results for its isothermal oxidation at 800 °C and 1200 °C. The discussion firstly considers the macrosegregation in the cast alloy, then its microstructure and the stability of phases, followed by the discussion of oxidation and the structures of the scales. The research breakthrough presented in this paper is the prevention of scale spallation at 1200 °C by the synergy of Ge and Sn with Al, Cr, Si, and Ti in the alloy OHS1.

## 2. Experimental

The alloy was prepared in the form of small buttons in a Ti-gettered Ar atmosphere using arc melting with a non-consumable tungsten electrode. The charge of pure elements (≥ 99.9 wt.% purity) was placed in a water-cooled copper crucible and was melted five times. For the heat treatment (1400 °C/100 h, consistent with the conditions used for the alloys KZ5, ZX8, and ZF6 [33,34,42]), the alloy was wrapped in Ta foil and placed in an alumina crucible in a calibrated alumina tube furnace where it was heat-treated under a constant flow of Ti-gettered Ar.

The microstructures were characterized using scanning electron microscopy (SEM) and powder X-ray diffraction (XRD). For the former, Jeol JSM 6400 SEM and Inspect F FEG SEM were used. The microstructures were studied in back-scattered electron (BSE) mode with EDS (energy dispersive spectrometry) microanalysis of the alloy and phases. EDS standardization was performed using, as standards, specimens of high-purity Nb, Ti, Cr, Si, Al, Ge, Sn, Co, and Al_2_O_3_ that were polished to 1-μm finish. The EDS was calibrated prior to analysis with the Co standard. At least five large-area analyses were performed in the top, bulk, and bottom of the button, and at least 10 analyses were obtained from each phase with size ≥5 μm to determine actual compositions.

For the identification of phases in the bulk of the alloy, a Siemens D500 diffractometer with CuKα radiation (λ = 1.540562 Å), 2θ from 20°–120°, and a step size of 0.02° was used. For the characterization of the surfaces of the oxidized alloy, we used glancing-angle XRD (GXRD) with a Siemens D5000 diffractometer with Cu K_α1_ and K_α2_ radiation (λ = 1.54178 Å), 2θ from 10°–100°, and a step size of 0.02°. Peaks in the diffractograms were identified by correlating data from the experiments with that from the JCPDS data (International Center for Diffraction Data). The scan type used for GXRD was detector scan, while that for bulk specimens was locked coupled. Prior to GXRD experiments, the glancing angle γ was selected with the aid of the AbsorbDX software, which evaluates the X-ray penetration depth for particular glancing-angle conditions.

The isothermal oxidation of the alloys was studied at 800 and 1200 °C for 100 h using a Netzsch STA F3 TG/DSC analyzer with an SiC furnace, with air flow rate of 20 mL/min, and with heating and cooling rates of 3 °C/min. Cubic specimens of size 3 × 3 × 3 mm^3^ and polished to 800-grit SiC finish were used for the thermogravimetry (TG) experiments. Weight change data such as those reported here were analyzed by using the equation ln(Δw) = lnK + nlnt, where Δw = Δm/A is the weight change per unit area, K is the reaction rate constant that embodies the sum of reaction rates, Δm is the weight change, A is the surface area before exposure, t is the oxidation time, and n is a constant. The oxidation reaction kinetics was then characterized as linear or parabolic depending on the value of n. If there was more than one mechanism involved, the corresponding section was evaluated to determine the oxidation kinetics from the equation $\Delta {w\;=\;k}_{l}\cdot t$ for linear oxidation and ${\left(\Delta w\right)}^{2}{\;=\;k}_{p}\cdot t$ for parabolic oxidation, where k_l_ is the linear rate constant and k_p_ is the parabolic rate constant [43].

## 3. Results

### 3.1. As-Cast Alloy

**Figure 1:**
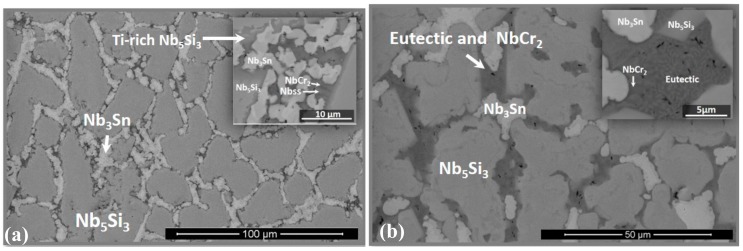
Back-scattered electron (BSE) images of the microstructure of OHS1-AC (as-cast): (**a**) bulk and (**b**) bottom.

The actual composition (at.%) of the as-cast alloy (OHS1-AC) was Nb-23Ti-18.5Si-4.4Cr-4.7Al-5Ge-4Sn. This was the average of all the analyses taken from the top, center, and bottom of the button. The average Sn concentration was lower than the nominal one owing to loss of Sn via evaporation during melting. The macrosegregation of Si was stronger than that of Ti. There was also weak macrosegregation of Al, Cr, and Sn. The typical microstructures are shown in Figure 1.

**Figure 2:**
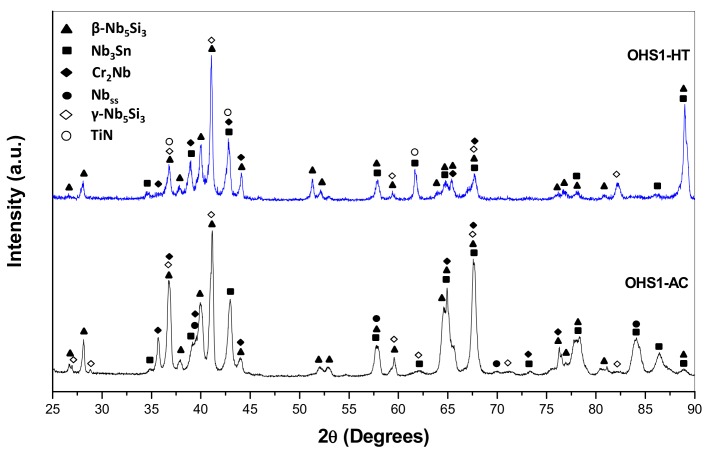
X-ray diffractograms (XRD) of the alloy OHS1 in the as-cast (AC) and heat-treated (HT) conditions. The XRD data agreed with the data from the following JCPDS (International Center for Diffraction Data) cards: (34-370) for Nb_ss_, (30-0875) for βNb_5_Si_3_, (65-4327) for Ti-rich Nbi_5_Si_3_, (19-875) for the Nb_3_Sn, (47-1637) for the NbCr_2_ Laves phase, and (38-1420) for the TiN.

According to the XRD data (Figure 2), the phases present in the microstructure were the Nb_ss_, C14-NbCr_2_, Nb_3_Sn, βNb_5_Si_3_, and γNb_5_Si_3_. The primary Nb_5_Si_3_ had Ti segregation at its boundaries (Ti-rich Nb_5_Si_3_). The Nb_3_Sn exhibited bright contrast, was observed in the inter-dendritic areas, and also had Ti-rich regions that exhibited dark-gray contrast. The C14-NbCr_2_ Laves phase exhibited the darkest contrast. The chemical compositions of the phases are given in the Table 1. These are the averages of all the analyses taken from the top, center, and bottom of the button.

The Nb_ss_ and C14-NbCr_2_ Laves phase were present at low volume fractions and were observed in the areas of dark contrast, where it was not possible to observe grain or phase boundaries. The analysis data for the Laves phase is in agreement with that from previous research [15,44]. It is likely that the analysis data of the Nb_ss_ is not accurate owing to the size of this phase. The Nb_ss_ was rich in Ti, with the concentrations of the latter in agreement with previous research [42,45,46], but unusually rich in Cr, the concentration of which was higher than the maximum solid solubility of Cr in Nb (about 24 at.%). According to previously published data about the Nb_ss_, for the Ti concentration given in the Table 1, the Cr content should be about 15 at.% [45]. The high Si concentration in the Nb_ss_ was out of step with most of the data in the literature but in agreement with the data for the Nb_ss_ in Ta containing Nb-24Ti-18Si-5Al-xCr-6Ta alloys (x = 5, 8) [46]. The solid solubility of Si in Nb_ss_ is, in general, higher in Sn- or Ge-containing Nb-24Ti-18Si-based alloys [32,33,34], compared with alloys without these two elements. The solubility for Ge or Sn in the Laves phase was very low and slightly lower than that in alloys with only Sn [32,33] or Ge [34,40] additions.

The microstructure in the top of the button was finer than that in the bulk, and it was more homogeneous in the bottom compared with the bulk and top. In the bottom, the same phases were present (Figure 1b), but with a higher volume fraction of the dark-contrast regions compared with the top and bulk of the button. In addition, a eutectic was observed in areas of black contrast. The average composition of the eutectic was 23.5Nb-33.5Ti-5.2Si-25.1Cr-7.7Al-1.8Ge-3.2Sn with Si + Al + Ge + Sn = 17.9 at.%, a value that is close to that of the Nb + Nb_3_Si eutectic in the Nb–Si binary [47].

The scrutiny of the eutectic was not easy owing to its fine structure. It is not possible to exclude the presence of the eutectic in the dark contrast areas in the top and bulk, where the average composition of the “eutectic” was 23.2Nb-27.5Ti-6.8Si-32Cr-6.7Al-1.3Ge-2.5Sn, considerably richer and poorer in Cr and Ti respectively, compared with the eutectic in the bottom, but with essentially the same Al + Si + Ge + Sn content (17.3 at.%). In the bottom of the button, the concentrations of elements in the eutectic were in the ranges (22.7–24)Nb-(30.6–35.9)Ti-(4.6–5.8)Si-(22.3–26.9)Cr-(7.2–8.3)Al-(1.5–2)Ge-(2.7–3.5)Sn, with the highest values giving Al + Si + Ge + Sn ≈ 20 at.%. The latter is close to the composition of the metastable Nb_ss_ + βNb_5_Si_3_ eutectic [48].

### 3.2. Heat-Treated Microstructure

**Figure 3:**
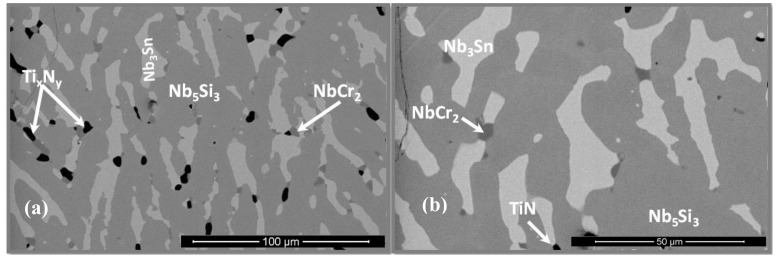
BSE images of the microstructure of OHS1-HT: (**a**) bulk and (**b**) bottom.

The actual composition of the heat-treated alloy (OHS1-HT) was Nb-24Ti-18Si-4.4Cr-4.7Al-5.1Ge-4.2Sn. The same phases were present as in the as-cast alloy with the exception of the solid solution (Figure 2). The microanalysis data are given in Table 2. These are the averages of all the analyses taken from the top, center, and bottom of the button. Weak chemical inhomogeneity of Si was still present. Figure 3 shows the typical microstructure. Detailed study of OHS1-HT in the SEM using microanalysis did not confirm the presence of Nb_ss_, in agreement with XRD (Figure 2). In the Nb_5_Si_3_ matrix, there were coarsened and elongated grains of Nb_3_Sn that in some areas were connected with the Laves phase. Contamination of the alloy by nitrogen resulted in the formation of TiN that exhibited black contrast. The Ti content in the Nb_3_Sn was essentially unchanged, but the Si + Al + Ge + Sn concentration increased to 23.4 at.%. In the Nb_5_Si_3_, the Ti concentration increased and the Si + Al + Ge + Sn concentration decreased slightly compared with the as-cast alloy (Table 1 and Table 2). No evidence of the prior eutectic microstructure was found in OHS1-HT.

### 3.3. Oxidation

**Figure 4:**
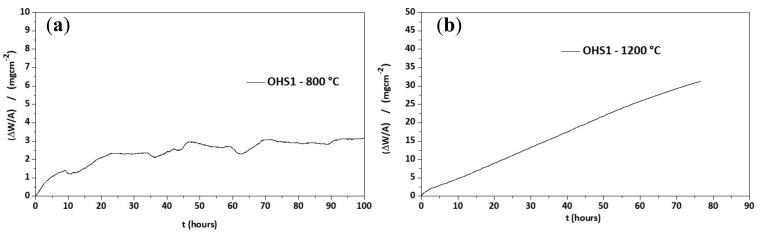
Thermogravimetry (TG) plots of the alloy OHS1 for isothermal oxidation in air at (**a**) 800 and (**b**) 1200 °C.

The alloy did not pest at 800 °C and formed an uneven thin adherent scale. There was no spallation of the scale at 1200 °C, where the specimen was completely covered by a light-brown scale. The isothermal oxidation data are shown in Figure 4, and the oxidation kinetics data are given in Table 3. For the isothermal oxidation at 800 °C, the n value was 0.46, meaning that the oxidation was parabolic. The decrease and increase in weight shown by the TG data in Figure 4a would suggest that the scale that formed at 800 °C was not stable. The alloy showed improved oxidation at 800 °C compared with linear oxidation of the alloy KZ5 which gained 30 mg/cm^2^ in weight after 85 h at 800 °C [23]. The oxidation at 1200 °C was described by a parabolic mechanism during the first 3.1 h, followed by a linear mechanism until 76.7 h, when the experiment stopped. The n = 0.83 value would suggest that overall the oxidation was linear.

### 3.4. The Scale at 800 °C

**Figure 5:**
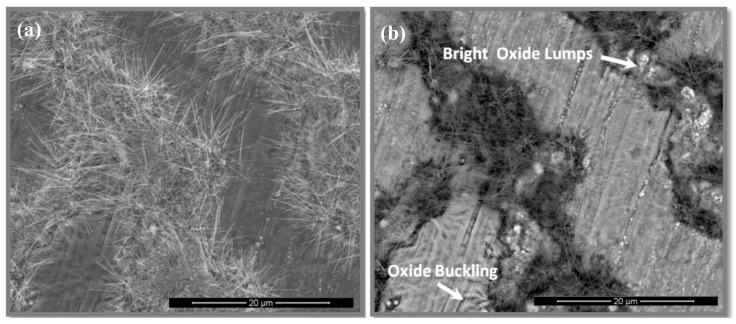

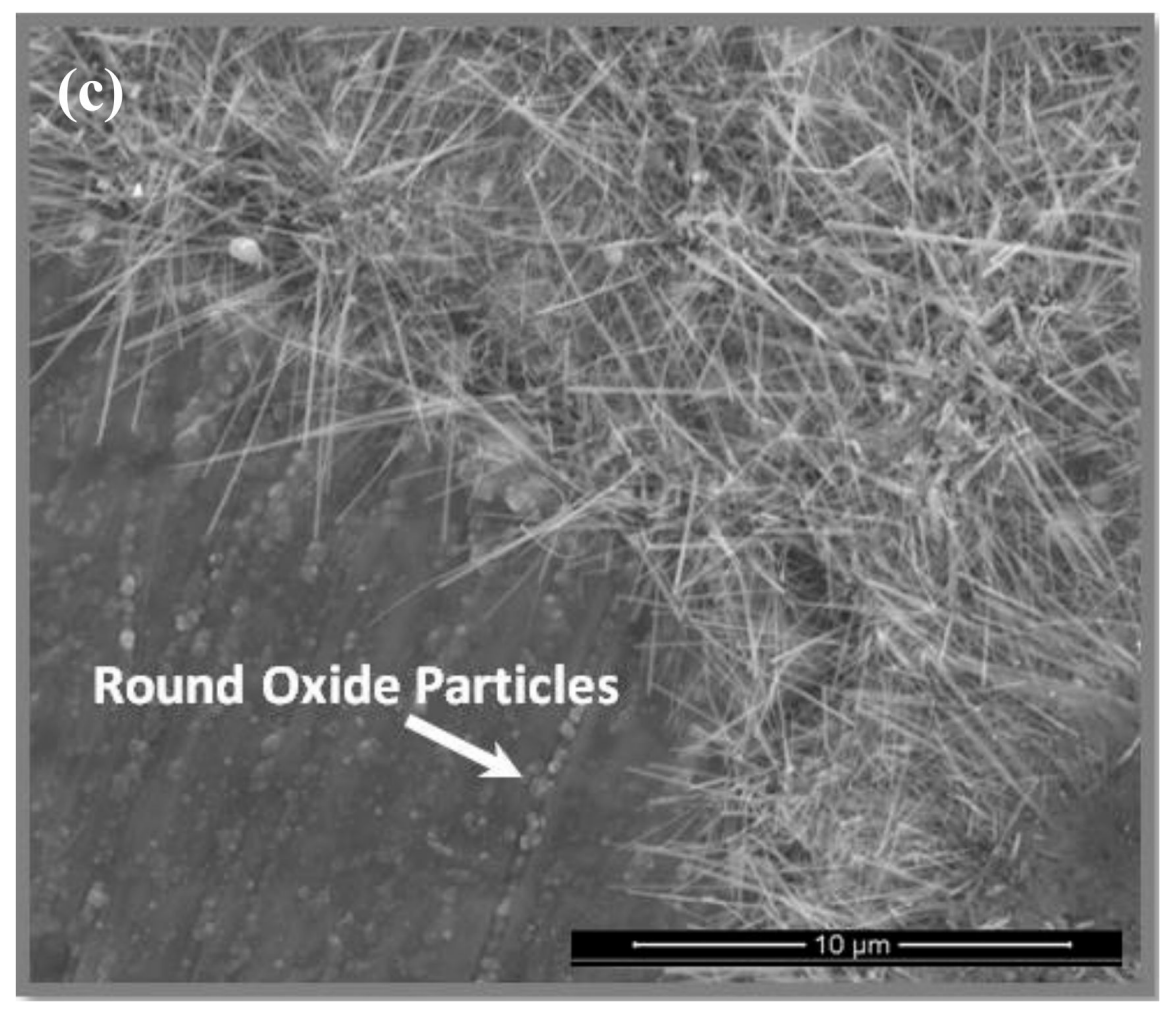
Images of scale surface of the alloy OHS1 after isothermal oxidation in air at 800 °C: (**a**) secondary electron (SE) image, (**b**) BSE image of the same region, and (**c**) SE image at higher magnification.

Three main features were observed in the scale surface, namely, a flat oxide with small round oxide particles that mostly formed in the polishing marks, oxide whiskers, and bright lumps that developed around the oxide whiskers (Figure 5). Figure 5a,b show the scale surface under secondary electron (SE) and BSE imaging conditions, respectively. The scale on top of the Nb_5_Si_3_ silicide was thin. However, the apparently adherent scale presented some buckling near the grain or phase boundaries (Figure 5b). The length of the oxide whiskers was about 10 µm.

**Figure 6:**
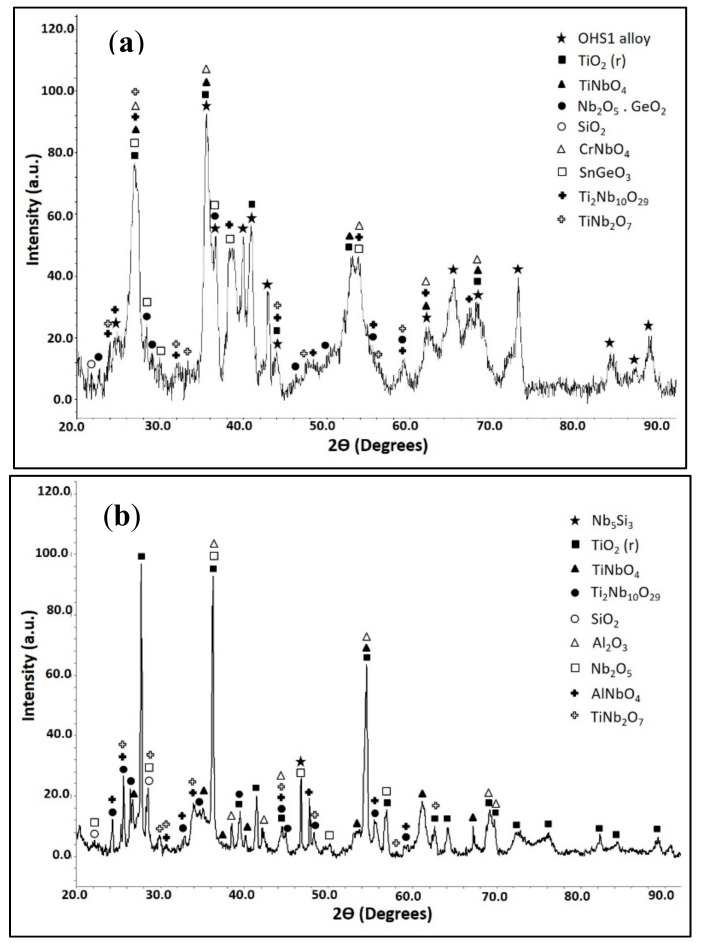
Glancing-angle XRD (GXRD) data of the scales formed on the alloy OHS1 at (**a**) 800 and (**b**) 1200 °C in air, in (**a**) γ = 2° and (**b**) γ = 10°. (**a**) TiO_2_ (JCPDS 21-1276), TiNbO_4_ (JCPDS 81-911), Nb_2_O_5_.GeO_2_ (JCPDS 19-0908), SiO_2_ (JCPDS 39-1425), CrNbO_4_ (JCPDS 81-909), SnGeO_3_ (32-0413), Ti_2_Nb_10_O_29_ (JCPDS 73-0242), and TiNb_2_O_7_ (JCPDS 77-1374); (**b**) TiO_2_ (JCPDS 21-1276), SiO_2_ (JCPDS 39-1425),Ti_2_Nb_10_O_29_ (JCPDS 73-0242), TiNb_2_O_7_ (JCPDS 77-1374), rutile type TiNbO_4_ (JCPDS 81-911) and AlNbO_4_ (JCPDS 41-0347), Nb_2_O_5_ (JCPDS 28-0317), and Al_2_O_3_ (JCPDS 70-3319).

**Figure 7:**
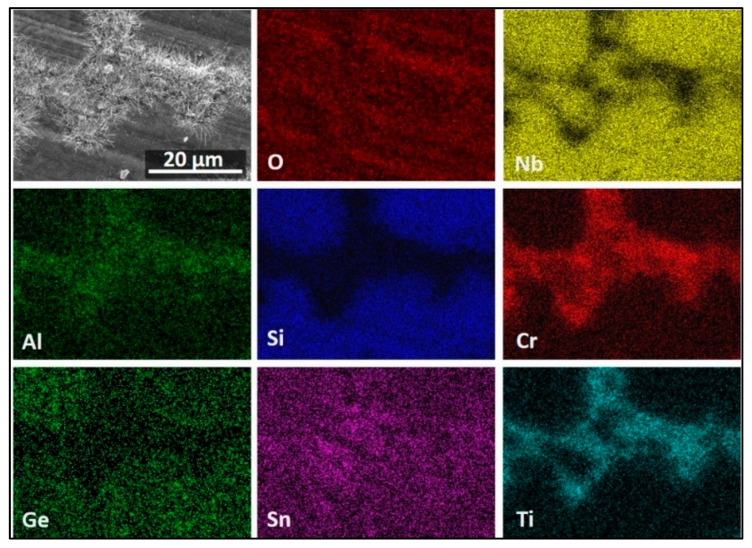
SE image and X-ray elemental maps of the scale surface of the alloy OHS1 after isothermal oxidation in air at 800 °C.

The GXRD data indicated (a) that the scale was composed of TiO_2_ (tetragonal, P42/mnm) TiNbO_4_ (tetragonal, I-4m2), Nb_2_O_5_.GeO_2_ (pseudo orthorhombic), SiO_2_ (tetragonal, P4_1_2_1_2), CrNbO_4_ (tetragonal, P42/mnm), SnGeO_3_ (monoclinic, P2/c), Ti_2_Nb_10_O_29_ (orthorhombic, Amma), and TiNb_2_O_7_ (monoclinic, C2/m) (Figure 6a), (b) that there were different titanium niobates formed as a result of the reaction of TiO_2_ with niobium oxides depending on their availability in different regions, and (c) that peaks from the Nb_3_Sn and βNb_5_Si_3_ phases were present in the substrate. The X-ray elemental maps (Figure 7) confirmed that the flat oxide grew on top of Nb_5_Si_3_ while the whiskers and the bright oxide lumps were formed over the area in between the Nb_5_Si_3_ grains, which consisted of Nb_3_Sn, NbCr_2_, and Nb_ss_. The oxide grown on top of Nb_5_Si_3_ was rich in Ge and Sn.

**Figure 8:**
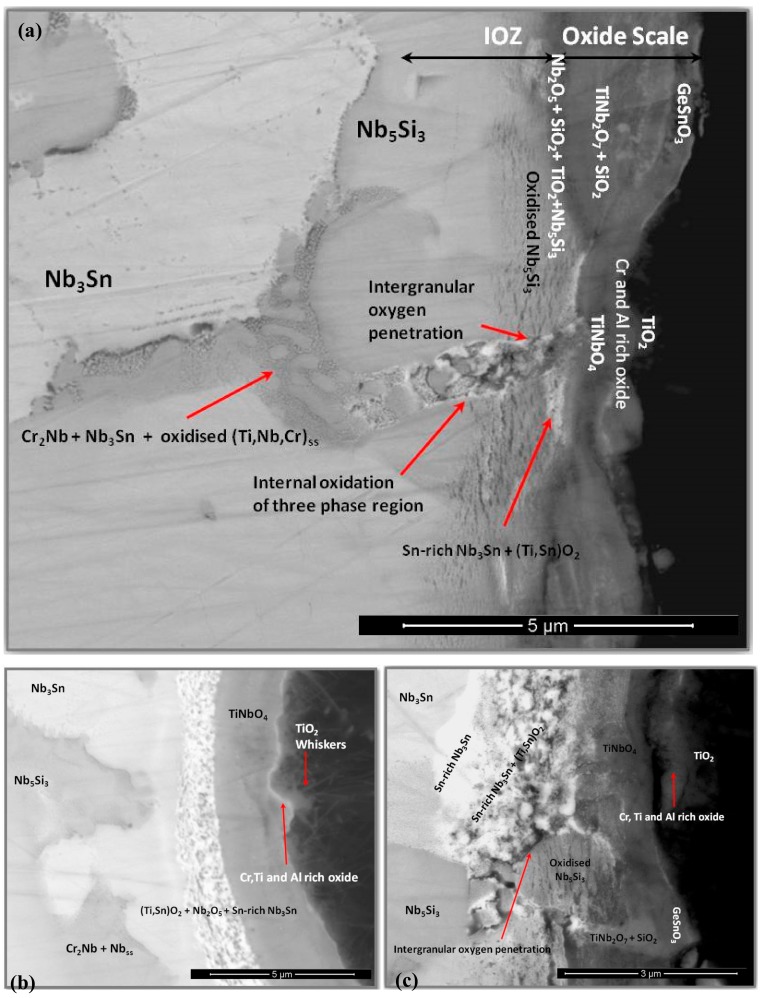
BSE images of a cross section of the alloy OHS1 after isothermal oxidation at 800 °C showing the microstructure (**a**) of the substrate and scale, (**b**) of the scale formed on top of the Nb_3_Sn phase, and (**c**) of the three-phase region after oxidation.

**Figure 9:**
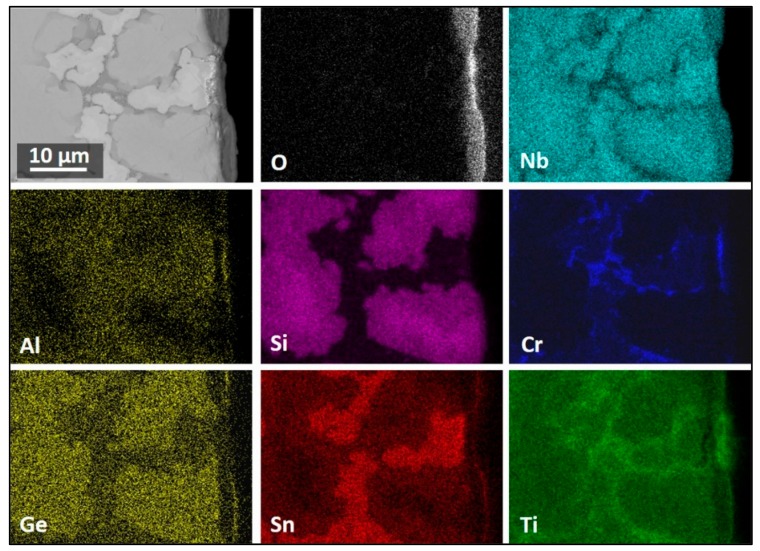
BSE image and X-ray elemental maps of a cross-section of the alloy OHS1 after isothermal oxidation in air at 800 °C.

The cross-section of the oxidized specimen in Figure 8 shows a scale about 2–5 µm thick, and an internal oxidation zone (IOZ) up to 3 µm deep. Some areas of the edge of the specimen presented large cracks parallel to the scale surface suggesting embrittlement; however, this was not common in the specimen. Figure 8 also shows that the microstructure of the scale depended on the oxidized phase. The microanalysis of the oxidized Ti-rich areas (composed of C14-NbCr_2_ and Nb_ss_) was strongly affected by the oxidation of the Nb_3_Sn, which was present in the same regions at a higher volume fraction. Apparently, the crisscrossed oxide whiskers were grown from the Ti-rich areas covering some oxide lumps. In the scale, the inter-granular areas presented very particular microstructures. The Nb_5_Si_3_ presented a very compact oxide layer in the outermost part of its scale (Figure 8 and Figure 9). According to the chemical analysis, the oxide was rich in Ge and Sn, suggesting that it could be the SnGeO_3_ oxide whose presence was suggested by the GXRD. The inner part of the scale in these areas was composed of SiO_2_ and different Ti_x_Nb_y_O_z_ compounds, with TiNb_2_O_7_ being the most common. The GXRD and qualitative chemical analyses also suggested the presence of the Ti_2_Nb_10_O_29_ compound and a decrease in Nb content toward the scale surface.

The microanalyses confirmed that the Nb_3_Sn formed a multi-layered complex oxide that consisted of at least three oxide layers; the outermost part contained TiO_2_ whiskers and (Sn,Ti)O_2_ lumps, then underneath was a Ti-, Cr-, and Al-rich oxide layer, and just below another oxide layer that was mainly composed of TiNbO_4_. Some areas of this layer also contained CrNbO_4_, SiO_2_, and perhaps some AlNbO_4_ resulting from the oxidation of the Al-containing Laves phase that was present in the vicinity (Figure 8b). The substrate/scale interface was characterized by the internal oxidation of the Nb_3_Sn and the formation of (Sn,Ti)O_2_ precipitates. The average composition (at.%) of these areas was 45O-27Nb-9Ti-10Sn-2Si-1Cr-4Al-2Ge. Just below the IOZ the composition of the Nb_3_Sn was 44.6Nb-27.2Ti-9.7Sn-3Si-6.9Cr-6.6Al-2Ge.

The scale that formed over the areas where the microstructure consisted of the Nb_ss_, C14-NbCr_2_ Laves, and Nb_3_Sn phases was composed of mixed oxides and a convoluted oxide with a very irregular microstructure and dark-contrast grain boundaries (Figure 8a,c), which would suggest that these areas served as the path for the diffusion of oxygen. Around the dark contrast inter-granular areas, multiple micro-cracks were formed on the Nb_5_Si_3_, which were parallel to the oxide surface (Figure 8a,c).

In the IOZ, the Ti-rich Nb_5_Si_3_ presented very small cracks parallel to the specimen surface. These cracked regions extended up to about 3 µm depth. The concentrations of Nb and Ti in Nb_3_Sn were reduced, and this compound was oxidized with dark oxide particles dispersed within some of its grains indicating that its oxidation depended on a critical Ti concentration, as the Ti-rich Nb_3_Sn was not contaminated by oxygen (Figure 8c). Oxygen could have diffused faster through the Nb_ss_ that was in the inter-dendritic areas, as dark-contrast particles were observed at different depths. The oxygen penetration depth was mostly associated with the extension of the cracked Nb_5_Si_3_ regions, with the cracking of the latter caused by the stresses generated by the oxidation of the Nb_ss_. The Laves phase in the IOZ was not oxidized.

The surface of the scale that formed on top of the Nb_5_Si_3_ phase was enriched in Ge and Sn in the outermost part, which is consistent with the high Ge and Sn contents observed in the elemental maps in Figure 7. Toward the inner part of the scale, the main components were the O, Nb, and Ti from titanium niobates. The oxide formed on top of the Nb_5_Si_3_ was rich in Si. The oxide formed on top of the three-phase region was rich in Ti and O. In these areas, the whiskers formed. The oxide below the whiskers was rich in Cr, Al, and Ti, followed by an oxide layer rich in O, Nb, and Ti with minimal presence of Ge (Figure 9). The Nb_3_Sn at the substrate/scale interface was richer in Sn compared with the rest of the alloy.

### 3.5. The Scale at 1200 °C

**Figure 10:**
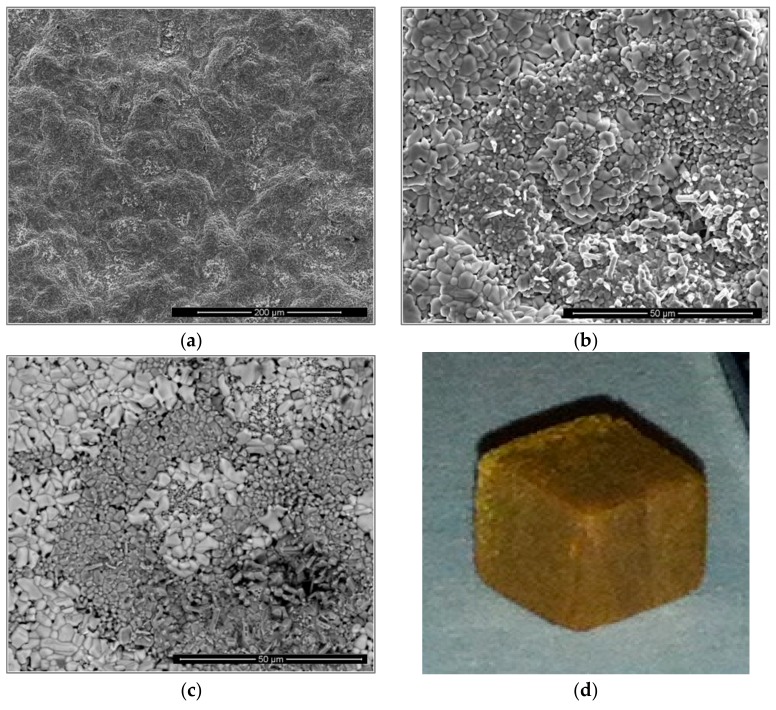
(**a**–**c**) Images of the surface of the scale formed on the alloy OHS1 after isothermal oxidation in air at 1200 °C and (**d**) the oxidized specimen. (**a**) and (**b**) are SE images, and (**c**) is a BSE image.

After the isothermal oxidation in air at 1200 °C, there was no scale spallation; instead, the specimen was covered by a light-brown scale with well-defined edges and some long cracks in the scale (Figure 10d). Typical images of the surface of the scale are shown in Figure 10. The SE image (Figure 10a) shows a continuous and bulged scale with some small cracks. The images in Figure 10b,c show the surface at higher magnification and it is possible to observe particles with different morphology and some porosity. The scale consisted of different oxides (Figure 10c). There were coarse and bright oxide particles, small and dense gray oxide particles, and gray protruding rods.

The GXRD data in Figure 6b indicated that the scale consisted of binary and ternary oxides, namely, TiO_2_ (tetragonal, *P*4_2_/mnm), SiO_2_ (tetragonal, P4_1_2_1_2), Ti_2_Nb_10_O_29_ (orthorhombic, Amma), TiNb_2_O_7_ (monoclinic, C2/m), TiNbO_4_ (tetragonal, *P*4_2_/mnm), AlNbO_4_ (monoclinic, C2/m), Nb_2_O_5_ (pseudo hexagonal, P6/mmm), and αAl_2_O_3_ (trigonal, R3c). The GXRD data were compared with microanalysis data taken from the oxidized specimen, and the agreement was good. The highest selected glancing angle was sufficient to detect phases to 200 µm depth. It is likely that some phases at the substrate/scale interface and in the IOZ were undetected because of the thick scale.

**Figure 11:**
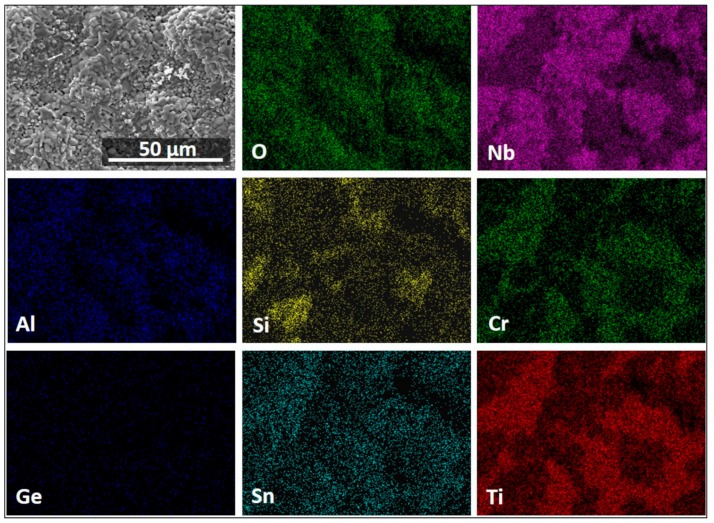
SE image and X-ray elemental maps of the scale surface of the alloy OHS1 after isothermal oxidation in air at 1200 °C.

The X-ray elemental maps in Figure 11 show an uneven elemental distribution in the scale surface. There was no Ge in the scale surface. The scale over the three-phase areas (NbCr_2_, Nb_ss_, and Nb_3_Sn) was rich in Cr, Ti, Al, and Sn. Figure 11 also shows TiO_2_ in the form of coarse whiskers or rods on top of the three-phase region where there was also Nb, Cr, Al, and Sn. However, the latter could be from oxides formed right below the oxide rods, as it was observed in the scale that formed at 800 °C. There were Nb oxides on top of the Nb_5_Si_3_ with low Si on the surface of the oxide. There was high Nb content in all the oxides, but the Nb concentration decreased when the oxides were rich in Ti, Cr, Al, and Sn.

**Figure 12:**
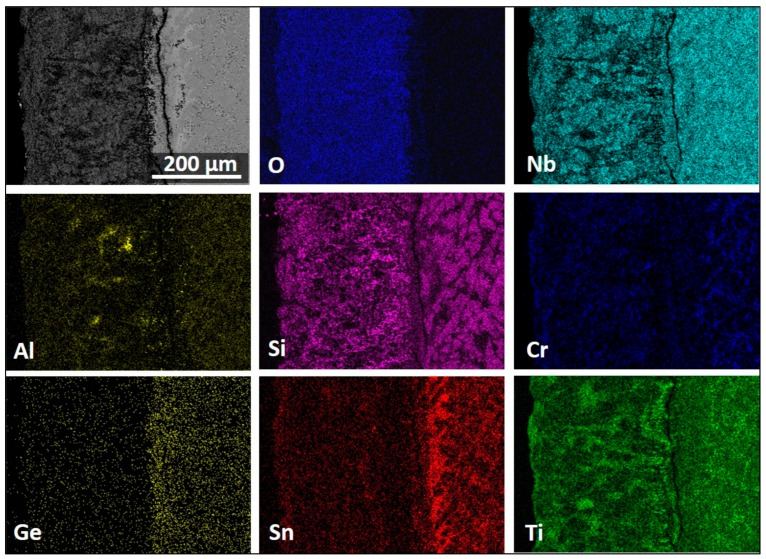
BSE image and X-ray elemental maps of a cross-section of the alloy OHS1 after isothermal oxidation in air at 1200 °C.

The X-ray elemental maps from a cross-section of the specimen are shown in Figure 12, showing the elemental distribution in the scale, the diffusion zone (DZ), and the substrate. The diffusion zone (DZ) includes the internal oxidation zone (IOZ). We refer to IOZ only when referring to regions where oxygen or nitrogen was present below the scale; otherwise, the term DZ is used.

Figure 12 shows a high Nb, Si, and Ti presence in the scale. The Nb was mostly found in the Ti_2_Nb_10_O_29_ and (Cr,Ti)NbO_4_ oxides, in agreement with the GXRD data. Low-Nb-content regions near the scale/substrate interface were rich in TiO_2_ or SiO_2_. Chromium was found mostly dissolved in the TiNbO_4_ oxide with higher content at the scale surface. The IOZ was depleted in Cr. There was Si present all over the scale in glassy SiO_2_ and dissolved in (Ti,Cr)NbO_4_. The Al content was practically the same in most of the scale and in the substrate; however, in some parts of the scale, the AlNbO_4_, (Cr,Al)NbO_4_ and Al_2_O_3_ around Ti-rich oxides presented a higher Al content. The scale did not contain Ge but had some Sn in it, and the IOZ presented a considerable enrichment of these two elements, particularly Sn, with a considerable reduction of Cr and Ti contents in these areas.

Microanalyses revealed that the Ge- and Sn-rich regions at the IOZ were the Nb_5_Ge_3_, (Ti,Nb)_6_Sn_5_, and Nb_5_Sn_2_Si phases, although peaks of these compounds were not recorded in GXRD. These compounds were found in areas with strong enrichment (segregation) of Si and Sn. The composition of the (Ti,Nb)_6_Sn_5_ was 17.8Nb-33.0Ti-0.3Si-2.5Cr-3.1Al-1.8Ge-41.5Sn. The Nb_5_Sn_2_Si had fixed values for Nb and Ti, and its composition was Nb-20Ti-(11.6–17.9)Si-(2.6–3.4)Cr-(1.5–3.3)Al-(5.4–6.3)Ge-(11.8–18.1)Sn. The Nb_5_Ge_3_ presented a wide solubility range for Ti, Si, Ge, and Al. When its Ge content was 24.3 at.%, the Ti, Si, and Al contents were 11.3, 13.4, and 0.2 at.%, respectively, and the Nb concentration was 45.8 at.%. When the Ge content was 13.5 at.%, the Ti, Si, and Al concentrations were 34.1, 16.0, and 2.2 at.%, respectively, and the Nb content was 29.1 at.%.

**Figure 13:**
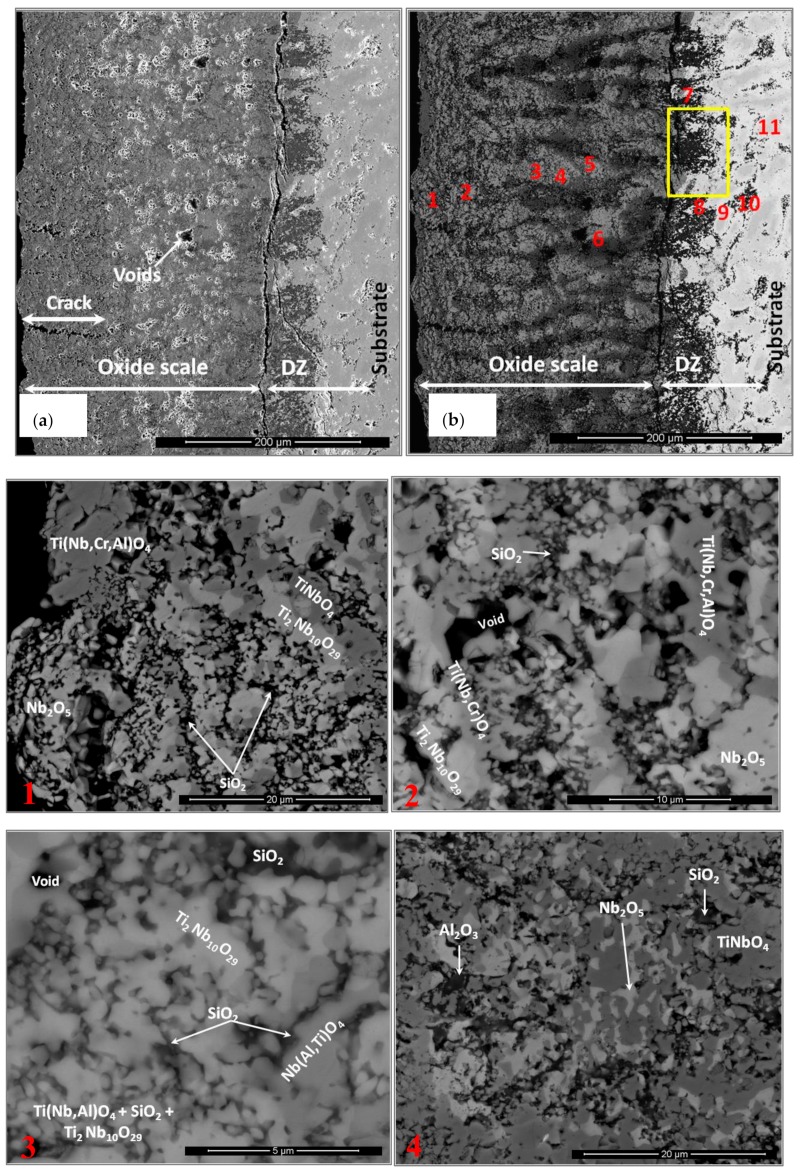

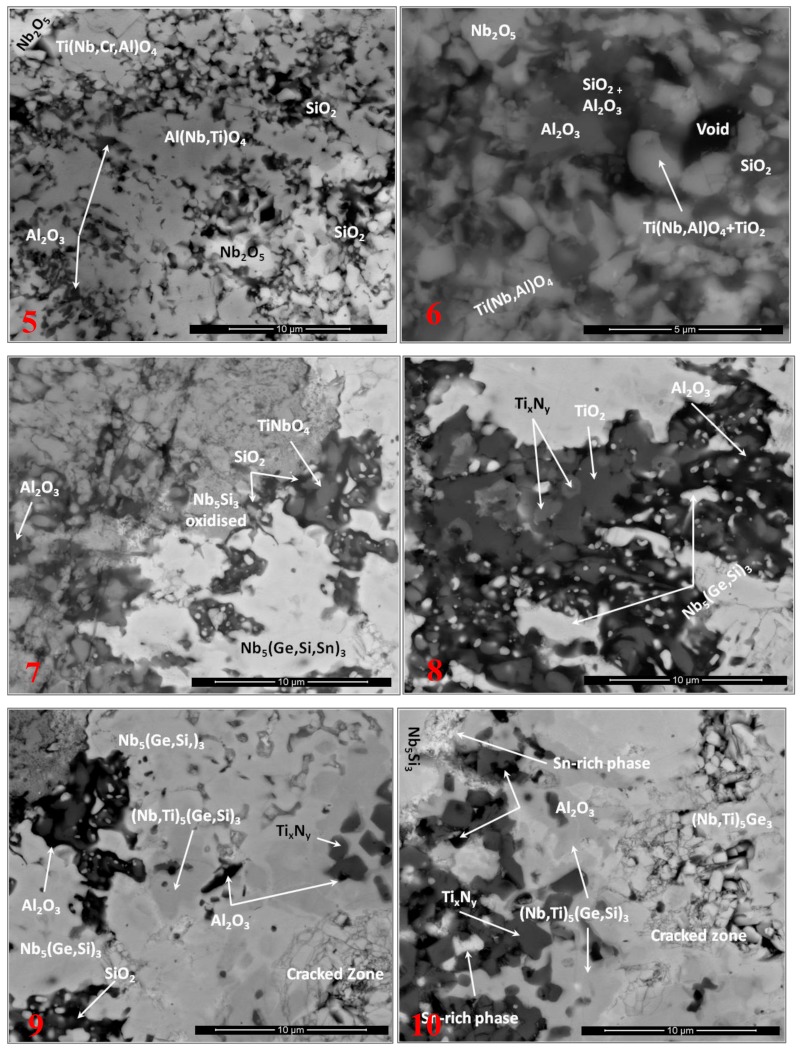

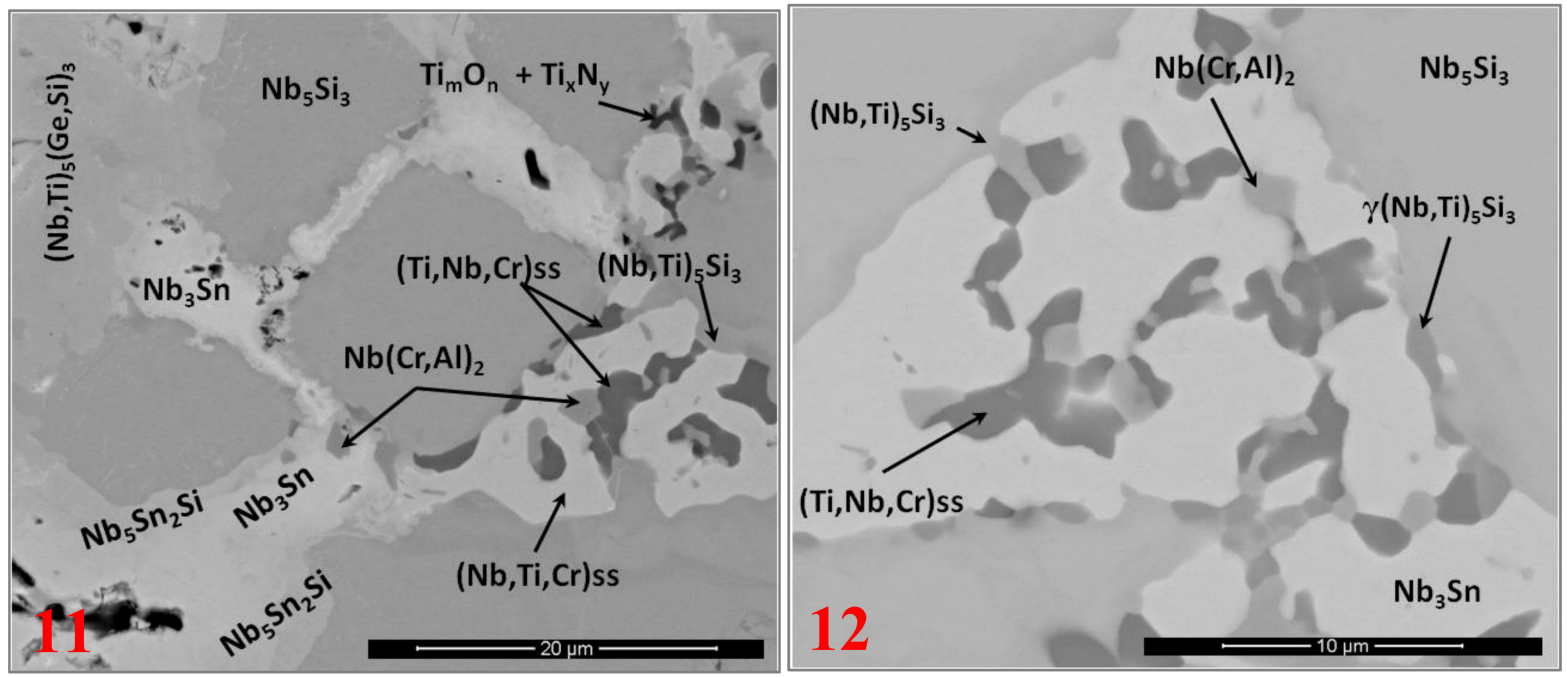
Images of cross-sections of the alloy OHS1 after isothermal oxidation in air at 1200 °C: (**a**) SE, (**b**) BSE. The red numbers are related to the following BSE images: (**c**) #1 oxide scale (OS), (**d**) #2 OS, (**e**) #3 OS, (**f**) #4 OS, (**g**) #5 OS, (**h**) #6 OS, (**i**) #7 diffusion zone (DZ), (**j**) #8 DZ, (**k**) #9 DZ, (**l**) #10 DZ, (**m**) #11 DZ, and (**n**) #12 bulk of the alloy after oxidation. For yellow box in (**b**), see Figure 14.

The cross-section images in Figure 13 show a very complex scale. Its thickness was about 300 µm, with a ~150-µm-thick diffusion zone (DZ) in which the IOZ was about 100 µm thick. The scale had porosity, voids, and cracks (Figure 13a). The latter were perpendicular to the surface and extended from the top of the scale to about 100 µm deep into the scale. There were also cracks parallel to the scale surface, but these were mostly found at the substrate/scale interface and did not affect the scale adherence. In the corners and regions near the corners of the specimen, the scale presented better adherence with no cracks.

The data in Figure 13 are consistent with the GXRD. There were at least three main contrasts related to the oxidation of the Nb_5_Si_3_ in the scale and, according to the microanalyses, SiO_2_ (black contrast), Ti_2_Nb_10_O_29_ (bright contrast), and TiNbO_4_ (dark-gray contrast). The TiNbO_4_ showed solubility for other elements; it had 2.1 to 5.05 at.% Cr and 1.85 to 4.58 at.% Al, while, in the Ti_2_Nb_10_O_29_, there was some Si solubility. However, the latter could be from its close proximity to SiO_2_. The oxidation of the Ti-rich areas presented oxides with the rutile crystal structure. The Al_2_O_3_, SiO_2_, and TiO_2_ oxides were also detected. They were dispersed in some regions within the scale and did not form a continuous scale. The internal oxidation of the alloy was observed along the grain boundaries of the Ti-rich areas.

**Figure 14:**
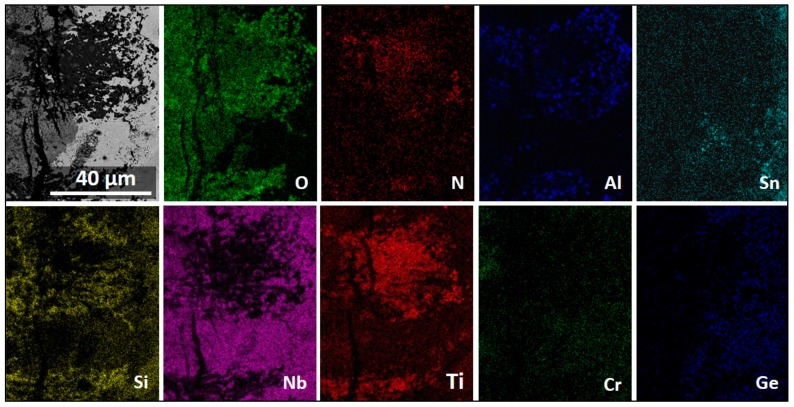
BSE image and X-ray elemental maps of a cross-section of the scale/substrate interface of the alloy OHS1 alloy after isothermal oxidation in air at 1200 °C. The selected area corresponds to the yellow box in Figure 13.

The numbered BSE images in Figure 13 show the scale microstructure in more detail. The phases were identified with microanalysis and were labeled accordingly. Images with numbers 1–6 correspond only to the scale, while images from 7–11 correspond to the DZ. Image 12 is from an area in the bulk of the alloy and shows that the bulk of the alloy was not oxidized. Figure 14 shows the elemental distribution in the selected area in Figure 13b (within the yellow rectangle). The maps revealed that Ti_x_N_y_ and TiO_2_ formed close to each other at the substrate/scale interface. Apparently, Al_2_O_3_, SiO_2_, and TiO_2_ were around the Ti_x_N_y_ compounds. 

The compositions of the phases in the bulk of the alloy were as follows: Nb-20Ti-21.8Si-2Cr-5.5Al-7.8Ge-3.2Sn for the Nb_5_Si_3_, Nb-22.8Ti-21.8Si-1.7Cr-4.4Al-6.9Ge-3.3Sn for the Ti-rich Nb_5_Si_3_, Nb-21.3Ti-1.8Si-4.4Cr-8.4Al-1.9Ge-10.8Sn for the Nb_3_Sn, and Nb-58.7Ti-7.8Si-12.1Cr-5.6Al-4.1Ge-1.6Sn for the solid solution, while the Laves phase was Nb-11.6Ti-8.1Si-47.5Cr-4.0Al-1.8Ge. The solid solution was very rich in Ti.

## 4. Discussion

### 4.1. Macrosegregation

In OHS1-AC, there was strong macrosegregation of Si and Ti, and weak macrosegregation of Al, Cr, and Sn. For Nb-silicide-based alloys, the macrosegregation of an element i is defined as C_max_^i^ − C_min_^i^, i.e., as the difference between the maximum and minimum concentrations of element i in the alloy. Macrosegregation of an element i is considered to occur when MACi = C_max_^i^ − C_min_^i^ > 2 at.% [49].

Tsakiropoulos [31] discussed the macrosegregation of elements in cast Nb-silicide-based alloys and linked the macrosegregation of Si with the partitioning of solutes between the key phases, namely, the Nb_ss_, Nb_5_Si_3_, C14-NbCr_2_ Laves, and Nb_ss_ + Nb_5_Si_3_ eutectic. Tin and Ge are two of the alloying additions that were found to have a strong effect on the macrosegregation of Si [32,33,34,40,41]. The ranking of Nb-silicide-based alloys in terms of increasing Si macrosegregation (MACSi) indicated that the latter tended to increase when the parameters ΔH_m_/T_m_ (“alloy entropy of fusion”), T_m_^sp^ (melting temperature of *sp* electronic configuration elements), and [ΔH_m_/T_m_][ΔH_m_^sd^/ΔH_m_^sp^]^−1^ increased, and when the ratios ΔH_m_^sd^/ΔH_m_^sp^ and T_m_^sd^/ T_m_^sp^ and the parameters ΔH_m_ (“alloy enthalpy of melting”), T_m_ (alloy melting temperature), and T_m_^sd^ (melting temperature of the *sd* electronic configuration elements) decreased [31].

In Table 4, the alloys KZ5, ZF6, OHS1, and ZX8 are ranked according to their MACSi values. The rational for using these data is as follows: the alloys (nominal compositions) KZ5 (Nb-18Si-24Ti-5Al-5Cr [42]), ZF6 (Nb-18Si-24Ti-5Al-5Cr-5Ge [34]), OHS1 (this work), and ZX8 (Nb-18Si-24Ti-5Al-5Cr-5Sn [33]) show the effects of Ge or Sn added individually and simultaneously in the alloy KZ5. The data for the parameters given in the Table 4 were calculated using the actual compositions of the as-cast alloys from References [33,34,42]. The data in Table 4 show no obvious trends between the parameters mentioned in the previous paragraph, when all four alloys were considered. However, if only the data for the alloys KZ5, ZF6, and OHS1 were considered, then the trends were clear (indicated by arrows), excluding the parameter ΔH_m_.

We can scrutinize how Al or Cr affects the macrosegregation of Si when they are in synergy with Sn or Ge individually and simultaneously, by considering the data in Table 5 and Table 6. In Table 5, the alloys (nominal compositions) KZ7 (Nb-18Si-24Ti-5Al [42]), ZF5 (Nb-18Si-24Ti-5Al-5Ge [41]), and ZX6 (Nb-18Si-24Ti-5Al-5Sn [33]) are compared with the alloy OHS1 in order to consider what the effects of Ge and Sn are when these elements are added individually or simultaneously in the “basis” alloy KZ7. The data for the parameters were calculated using the actual compositions of the cast alloys from References [33,41,42]. The data in Table 5 show that the aforementioned trends between the parameters were followed, with the exception of the parameter ΔH_m_, which would suggest (i) that Al, in synergy with Ge or Sn individually, increases the macrosegregation of Si, (ii) that Sn has a stronger effect than Ge, and (iii) that their combined effect is enhanced when Al, Ge, and Sn are present simultaneously in the alloy. What is the role of Cr? To answer this question, we need to consider Table 6.

In Table 6, the alloys (nominal compositions) KZ4 (Nb-18Si-24Ti-5Cr [42]), ZF4 (Nb-18Si-24Ti-5Cr-5Ge [40]), and ZX4 (Nb-18Si-24Ti-5Cr-5Sn [33]) are compared with the alloy OHS1 to consider what the effects of Ge and Sn are when these elements are added individually or simultaneously in the “basis” alloy KZ4. The data for the parameters were calculated using the actual compositions of the cast alloys from References [33,40,42]. The data in Table 6 show that the aforementioned trends between the parameters were followed for the alloys in which Ge or Sn were in synergy with Cr individually (red numbers and arrows in the table), again with the exception of the parameter ΔH_m_.

The data in Table 6 show that the synergy of Cr with Sn has a stronger effect on (increases) MACSi than that of Cr with Ge (compare KZ4 and ZX4 with KZ4 and ZF4). Furthermore, when the data for the alloy OHS1 were considered, the values of its parameters were “minimum” or “maximum” in the trends established by the synergy of Cr with Ge or Sn individually in the alloys. Table 5 and Table 6 confirm that, compared with Ge, Sn has a stronger effect on MACSi when it is in synergy with Al or Cr individually and that the strong effect of the synergy of Sn with Cr can be “controlled” by adding Ge to the alloy. Thus, the synergy of the alloying additions of Al, Cr, Ge, and Sn in Nb-silicide-based alloys is beneficial not only in terms of oxidation behavior, but also because it reduces the macrosegregation of Si.

### 4.2. Microstructure

Niobium can form the A15 Nb_3_Al, Nb_3_Ge, Nb_3_Sn, and Nb_3_Si compounds that have Cr_3_Si as a prototype [50]; thus, Al, Ge, Sn, and Si would be expected to stabilize the A15 structure. Vellios and Tsakiropoulos [51] showed that the Nb_3_Sn was stable in as-cast Nb-silicide-based alloys with 5 at.% Sn. This was confirmed by Xu et al. [33] and Knittel et al. [22]. In alloys with up to 5 at.% Ge addition, an A15 phase was not observed [34,40,41,52,53].

The Nb_5_Ge_3_ with prototype W_5_Si_3_ (i.e., isomorphous with βNb_5_Si_3_) is stable at all temperatures, and the Nb_5_Ge_3_ with prototype Cr_5_B_3_ (i.e., isomorphous with αNb_5_Si_3_) is not a stable phase in the Nb–Ge binary [54]. The Nb_ss_ + βNb_5_Si_3_ eutectic is metastable in the Nb–Si binary. In binary alloys, this eutectic is promoted by solidification at high cooling rates and/or high melt undercoolings (i.e., under RS (rapid solidification) conditions [55]), while, in ternary and higher-order alloys, it is also promoted by additions that can suppress the Nb_3_Si silicide [48]. Tin and Ge are such additions, as is Al, but not Cr when added to Nb-24Ti-18Si [32,33,34,40,41,42,52,53]. When Al and Cr were present simultaneously in Nb-24Ti-18Si-5Al-5Cr (alloy KZ5 in Reference [42]), the Nb_ss_ + βNb_5_Si_3_ was formed instead of the Nb_ss_ + Nb_3_Si eutectic. In the Nb–Si binary, the Si content of the metastable eutectic was suggested to be about 20 at.% [55] (although this value depends on which version of the Nb–Si binary phase diagram is used [48]). In ternary and higher-order alloys, the concentration of Si plus other element(s) (like Al, Sn, Ge) in the eutectic is about 21 at.% [48].

The microstructure of OHS1-AC would suggest that the primary phase was the βNb_5_Si_3_, which is also consistent with the higher melting temperature of this phase compared with the A15-Nb_3_Sn, the Nb_ss_, and the C14-NbCr_2_ Laves phase. The “architecture” of the microstructures of the as-cast alloys KZ5 [42], ZF6 [34], ZX8 [33] (see above for nominal compositions), and OHS1-AC was the same in that it consisted of primary βNb_5_Si_3_ with the other phases formed in between the primary silicide grains. In the alloy KZ5 (no Ge and Sn), the other phases were Nb_ss_ and Nb_ss_ + βNb_5_Si_3_ eutectic; in ZF6 (Ge present), the other phases were the Nb_ss_ and C14-NbCr_2_ Laves phase, and, in ZX8 (Sn present), the other phases were the A15-Nb_3_Sn and C14-NbCr_2_ Laves phase. This would suggest (i) that, in OHS1, the solidification path of the inter-dendritic melt “was defined” by the synergy of Sn with the other alloying elements, and (ii) that the partitioning of Ge to Nb_5_Si_3_ stabilized the βNb_5_Si_3_. In OHS1-AC, the eutectic was too fine to be characterized; it was rich in Cr and its average Al + Ge + Si + Sn content was about 17.9 at.% and about 20 at.% when the highest analysis values were considered. The Nb_3_Si silicide was not observed in OHS1-AC. The phases Nb_ss_ and βNb_5_Si_3_ participated in the eutectic; however, because of the high Cr content of the latter and evidence for the presence of NbCr_2_ in the eutectic (see insert in Figure 1b), it is suggested that the eutectic was a ternary one formed by the Nb_ss_, βNb_5_Si_3_, and the C14-NbCr_2_ Laves phase. As the primary βNb_5_Si_3_ formed, the surrounding liquid became richer in Ti, Cr, Al, and Sn and poorer in Si and Ge. In the latter liquid, the A15-Nb_3_Sn, the Nb_ss_, and the C14-NbCr_2_ Laves phase, as well as the eutectic, were formed. It is proposed that the solidification path of OHS1-AC was L → L + βNb_5_Si_3_ → L + βNb_5_Si_3_ + Nb_3_Sn → L + βNb_5_Si_3_ + Nb_3_Sn + Nb_ss_ → L + βNb_5_Si_3_ + Nb_3_Sn + Nb_ss_ + NbCr_2_ → βNb_5_Si_3_ + Nb_3_Sn + Nb_ss_ + NbCr_2_ + (Nb_ss_ + βNb_5_Si_3_ + NbCr_2_)_eutectic_.

In addition to the Nb_5_Si_3_ silicide, in the heat-treated alloys KZ5 [42] and ZF6 [34], the other “stable” phase was the Nb_ss_; in the alloy ZX8 [33], the other phases were the A15-Nb_3_Sn and the Nb_ss_, and, in OHS1-HT, the other phases were the A15-Nb_3_Sn and the C14-NbCr_2_ Laves phase. In the heat-treated alloys KZ7 (Nb-18Si-24Ti-5Al [42]) and ZX8 [33], the βNb_5_Si_3_ transformed to αNb_5_Si_3_; however, in the heat-treated KZ5, both silicides were present and, in ZF6-HT, there was weak evidence for αNb_5_Si_3_, although most of the XRD peaks corresponded to βNb_5_Si_3_, while, in the OHS1-HT, the βNb_5_Si_3_ was stable after the heat treatment. This would suggest that, in OHS1, the synergy of Ge and Sn with the other elements (i) stabilized the C14-NbCr_2_ Laves phase, (ii) destabilized the Nb_ss_, and (iii) stabilized the βNb_5_Si_3_ or made the βNb_5_Si_3_→αNb_5_Si_3_ transformation promoted by Al [42] or Sn [51] more sluggish.

### 4.3. Oxidation

#### 4.3.1. Oxidation at 800 °C

There were differences and similarities in the oxidation behavior at 800 °C of the alloys OHS1, ZX8, and ZF6. There was no pest oxidation, and all three alloys formed a thin adherent scale (about 8 and 5 μm thick in ZF6 and ZX8, respectively), which was slightly uneven in OHS1 (2–5 µm thick). The DZ in the latter was less deep (3 µm) compared with that in ZX8 (10 µm), and there was no DZ in ZF6 (i.e., the Nb_ss_ and Nb_5_Si_3_ below the scale/substrate interface were not contaminated by oxygen). Compared with the parabolic rate constants of the alloys ZX8 [33] and ZF6 [38], that of OHS1 was one order of magnitude higher, and the weight change was similar to the former but about three times that of the latter. The above data would suggest that, in the pest regime, the synergy of Ge with Al, Cr, and Ti was more efficient than that of Ge and Sn together.

At 800 °C, the substrate below the scale/substrate interface was richer in Sn in OHS1 and ZX8, and, in the former, the Laves phase in the IOZ was not oxidized. Comparison of the oxides that were determined by GXRD in the alloys ZF6 and OHS1 shows that CrNbO_4_ and TiNb_2_O_7_ were present in both alloys, the AlNbO_4_, Nb_2_O_5_, and GeO_2_ (hexagonal, P3_2_21) were present only in ZF6, and the TiO_2_, TiNbO_4_, SiO_2_, SnGeO_3_, Ti_2_Nb_10_O_29_ and Nb_2_O_5_·GeO_2_ were present only in OHS1.

In OHS1, the scale that formed on top of Nb_5_Si_3_ was thin and compact with some oxide buckling in areas of the surface that were above the microstructure between the βNb_5_Si_3_ grains (Figure 5) where thicker oxide was formed (Figure 8). Scale buckling is produced by the rise of internal stresses in the oxides forming the scale. The growth of voluminous oxide particles could cause scale buckling and cracking of the Nb_5_Si_3_ near the substrate/scale interface. Deeper oxygen penetration was found at the phase boundaries, which would suggest that they played a very important role in the inward transport of oxygen and the outward transport of reactants. The inter-granular areas were rich in Ti and would be expected to be more reactive than the Ti-poor areas. Brittle phases unable to cope with the fast rise of stresses induced by the growth of oxide particles cracked for relief, allowing further oxygen diffusion into the alloy. The high solubility of Ti, Cr, Si, Sn, and Al in the Nb_ss_ and oxygen anion diffusion in the alloy would embrittle the solid solution. It was not possible to confirm this using micro or nano-hardness testing because the areas with Nb_ss_ were too small.

The inherent brittleness of the Laves and Nb_3_Sn phases could also have contributed to cracking of the scale and could explain the cracks parallel to the oxide surface observed in some areas near to the substrate/scale interface. Oxide transformations also change the growth rate of oxide, whereby the growth of different oxides in some small areas could lead to stresses and cracks, particularly in the Nb_5_Si_3_ and Nb_3_Sn compounds. Keeping the above in mind, it was encouraging to see that there was no oxide spallation or specimen disintegration. The TiO_2_ whiskers seen in Figure 5 could have formed as a result of the oxidation of the Nb_ss_ which was very rich in Ti (also observed by Menon et al. [18]). According to Kofstad and Kjöllesdal [56], whiskers may form through a plastic flow mechanism caused from the growth of stresses in the oxide scale. At the substrate/scale interface, submicron (Sn,Ti)O_2_ rutile type oxide particles were observed within the Nb_3_Sn. These features were also observed in Nb-24Ti-18Si-5Cr-5Al-2Mo-5Hf-5Sn (alloy JG6 in Reference [20]).

Menon et al. [18] reported that the surfaces of alloys with Ge or Ge and Sn additions that were oxidized at T ≤ 900 °C were characterized by cracks parallel to the surfaces just below the oxide layer. They called this “a phenomenon of progressive failure” of Nb-silicide-based alloys during low-temperature oxidation, which they claimed to be distinct from pest oxidation. The formation of cracks in the substrate just below the substrate/scale interface and parallel to it was observed in oxidation in the pest regime in Nb-24Ti-18Si-based alloys without Sn or Ge additions [23,24], and with Sn addition [20,32,33] or Ge addition [38], in the alloys Nb-23Ti-5Si-5Al-5Hf-5V-2Cr-2Sn and Nb-30Ti-10Si-5Cr-5Sn-3Fe-2Al-2Hf [21] and in the alloys studied by Knittel et al. [22]. However, this “phenomenon” was essentially suppressed in the alloy ZF6 (Nb-24Ti-18Si-5Al-5Cr-5Ge) [38]. As oxidation progresses, the substrate below the scale/substrate interface becomes contaminated by oxygen, with the Nb_ss_ more severely affected than the Nb_5_Si_3_ [20,22,32,33,38,57] and other phases that might be present, for example, A15-Nb_3_Sn [15,33]. The volume changes and accompanying stresses that arise from the oxidation of the substrate below the said interface cause the low-toughness Nb_5_Si_3_ to crack. The data for alloys with/without stable Nb_ss_ would suggest that suppression of the contamination of the substrate by oxygen limits the “phenomenon of progressive failure”.

#### 4.3.2. Oxidation at 1200 °C

Spallation of the oxide scales that are formed on Nb-silicide-based alloys at T ≥ 1000 °C is common [18,22,23,24,25]. Different volumes of oxidation products cause internal stresses in the scale. Furthermore, internal stresses in the scale can arise from the anisotropy of the coefficients of thermal expansion (CTE) of oxides in the scale. Internal stresses contribute to the cracking of the latter and its separation from the substrate. Such stresses can be significant. For example, the internal stresses along the crystallographic directions a, b, and c of Nb_2_O_5_ during cooling from 1000 to 400 °C were estimated by Manning et al. [58] to be σ_a_ = 276 MPa, σ_b_ = −165 MPa, and σ_c_ = 283 MPa.

Compared with the alloys ZX8 [33] and ZF6 [38] that exhibited scale spallation at 1200 °C, the discovery that the specimen of OHS1 was covered by a continuous adherent scale at 1200 °C was exciting. Considering the parabolic and linear rate constants of the alloys ZX8 [33] and ZF6 [38], the former was lower by one order of magnitude for OHS1 and the latter was essentially the same for all three alloys. The weight change of OHS1 was the smallest, and that of ZF6 was the highest (but not very different from that of ZX8). The scale thickness of ZF6 was the highest, and those of ZX8 and OHS1 were the same. The above data would suggest that, in the high-temperature regime, the simultaneous presence of Ge and Sn with Al, Cr, and Ti in OHS1 was more effective than that of Ge or Sn individually with the same elements in the alloys ZX8 and ZF6.

The same Sn-rich compounds were formed in the IOZ below the scale/substrate interface in OHS1, as was the case in ZX8, plus (Ti,Nb)_6_Sn_5_. There was no evidence of the recrystallized structures that were observed in ZX8 [33], which would suggest that the strain energy in the substrate below the said interface was lower in OHS1 compared with ZX8. In the substrate below the scale formed on ZX8, there was an Sn rich phase (analysis number 12 in Table 13 in Reference [33]) that could be (Nb,Ti)_6_Sn_5_. This compound had a lower Ti concentration (14.2 at.%) than the (Ti,Nb)_6_Sn_5_ (33 at.%) that was observed in OHS1. The Ti_6_Sn_5_ compound is isomorphous with Nb_6_Sn_5_ [50], but has a significantly higher melting temperature (1490 °C) than the latter (916 °C). Thus, melting of the (Ti,Nb)_6_Sn_5_ compound in the substrate below the scale was not possible. In the IOZ in OHS1, there was also Nb_5_Ge_3_ alloyed with Al, Si, and Ti with wide solubility ranges of these elements and Ge. The latter was not observed in ZF6. The Nb_5_Ge_3_ has lower shear (G) and Young’s (E) moduli and higher Poisson’s ratio (ν) than βNb_5_Si_3_ [54,59], as well as G, E, and ν values closer to the values of Ti-alloyed βNb_5_Si_3_, particularly as the Ti concentration in the silicide increases [39]. Furthermore, in the IOZ of OHS1, the concentrations of Cr and Ti were reduced significantly in the areas that were enriched in Ge and Sn below the said interface.

#### 4.3.3. Oxide Scales

Nb oxide(s), rutile, and niobates are the typical oxides in the scales of Nb-silicide-based alloys [10]. Amorphous silicates were reported in MASC alloy scale during the early stages of oxidation at 1200 °C [60]. In Nb oxides and TiO_2_, crystallographic shear and point defects produce (assist) stoichiometry changes. The former can accommodate large oxygen deficiencies, keeping the metal ion coordination, while the latter cannot account for high variations in stoichiometry and can even be eliminated in the process of crystallographic shear [61].

The structure of rutile is slightly more open, parallel to the c axis. The diffusivity of O parallel to the c axis is larger than perpendicular to it [62]. Also, the thermal conductivity of rutile is fastest parallel to the c axis [63]. The Ti–O bond lengths and strengths are independent of crystallographic direction [64]. The diffusion mechanisms of both Ti and O in rutile are controlled largely by defects, and the O diffusion depends on the environment in which TiO_2_ grows [62]. The defect structure in rutile is dominated by O vacancies at low temperatures and high O pressures, and by Ti interstitials at high temperatures and low O pressures [65].

The scale that formed on OHS1 was rich in Nb, Si, and Ti, and it contained some Sn and Cr but no Ge. Comparison of the oxides that were determined by GXRD in the alloys ZF6 and OHS1 shows that the oxides Nb_2_O_5_, SiO_2_, TiNb_2_O_7_, and AlNbO_4_ were present in both alloys, the AlNbO_4_ and GeO_2_ were present only in ZF6 (as was the case at 800 °C), and the oxides TiO_2_, TiNbO_4_, and Ti_2_Nb_10_O_29_ were present only in OHS1 (as the case at 800 °C), while αAl_2_O_3_ was observed only in OHS1 at 1200 °C. In OHS1, some Cr was in solution in (Al,Cr)NbO_4_ and (Ti,Cr)NbO_4_. In some areas of the scale surface, there was a coarse and compact oxide with no Ge and Si content. This oxide was not continuous and was surrounded by different Ti niobates and SiO_2_.

In the substrate below the scale, the oxygen solubility in the Nb_5_Ge_3_ was higher compared with Nb_5_Si_3_, and TiO_2_ and SiO_2_ surrounded the former. The substrate below the scale/substrate interface was richer in Ge and Sn and poorer in Cr and Ti. Furthermore, compared with the chemical composition of the solid solution in the microstructure of the specimen used for isothermal oxidation, which was Nb-based and Ti-rich (Nb/Ti > 1), the solid solution in the bulk of the oxidized specimen was Ti-based, meaning that it was very rich in Ti with Nb/Ti < 1. This enrichment of the solid solution in Ti would have significantly improved its oxidation resistance [11].

Most of the Cr was found in MNbO_4_ oxide(s) in the surface of the scale. The Cr content in the scale increased from the substrate/scale interface toward the surface of the scale. No Cr oxide particles were found in the scale and no Cr solute in Al_2_O_3_. Ti and Cr probably affected the oxygen solubility in Nb and, thus, the formation of Nb_2_O_5_ was dramatically reduced allowing the formation of MNbO_4_ oxide(s). Indeed, as discussed by Hurlen [66] and Kofstad and Kjollesdal [56], the oxidation resistance of Nb at high temperatures is strongly influenced by its oxygen solubility, which could increase above saturation values under particular conditions. The starting microstructure of the oxidized specimen contained a low vol.% of the Nb_ss_ with Al, Cr, Ge, Sn, and Ti in solution. The oxidation of the Nb_ss_ depends on the concentration of these solutes [18,20,22,24]. For example, Ti is known to significantly improve the oxidation of Nb–Ti solid solutions [11,67].

The microstructures of the specimens of the alloys OHS1, ZX8, and ZF6 at the start of the oxidation experiments were different, with no Nb_ss_ in ZX8, no A15 compound in ZF6, and the βNb_5_Si_3_ and the C14-NbCr_2_ Laves phase present in all three alloys. In the heat-treated alloys, the Laves phase was stable in all three alloys, and the Nb_ss_ was stable in the alloys ZX8 and ZF6 but not in OHS1. At 800 °C, the alloys OHS1, ZX8, and ZF6 did not pest and formed thin adherent scales, compared with the alloys KZ5 (Nb-24Ti-18Si-5Cr-5Al, no pest, Maltese cross formation [23]) and KZ2 (Nb-24Ti-18Si-8Cr-4Al, no pest, spallation of thin scale [23]), the starting microstructures of which consisted of Nb_ss_, βNb_5_Si_3_, and αNb_5_Si_3_ in both alloys, plus the C14-NbCr_2_ Laves phase in the latter [23,42]. This would suggest that individually or simultaneously alloying with Ge or Sn is essential for the suppression of pest oxidation but not the presence of the A15-Nb_3_Sn in the bulk microstructure.

It is challenging to answer with certainty why the adhesion of the scale that formed on OHS1 at 1200 °C improved dramatically compared with the alloys ZX8 and ZF6. Similar to ZX8, in OHS1, there was enrichment in Sn in the substrate below the scale/substrate interface and formation of Sn-rich compounds (Nb_5_Sn_2_Si, Nb_3_Sn) with Ti nitride(s) and no Laves phase; however, unlike ZX8, in the OHS1, the (Ti,Nb)_6_Sn_5_ was also formed. In the latter alloy, there was also enrichment with Ge of the substrate below the said interface and formation of Nb_5_Ge_3_ with wide solubility ranges for Al, Ge, Si, and Ti. This was not observed in the alloy ZF6. Furthermore, the typical oxides that form in the scales of Nb-silicide-based alloys [10] were also observed in the scales of OHS1, ZX8, and ZF6; however, there were differences between individual scales (see above).

In the alloy ZF6, both AlNbO_4_ and CrNbO_4_ were formed, while only the former was formed with Cr in solution in OHS1, which means that more Cr was present in the scale of ZF6 because there is incomplete solid solubility between AlNbO_4_ and CrNbO_4_ [68]. Indeed, up to some critical Cr content, the (Al,Cr)NbO_4_ is one phase with the structure of AlNbO_4_ (monoclinic, C2/m space group) compared with two phases AlNbO_4_ plus CrNbO_4_ (tetragonal (rutile type structure), P42/mnm) when the critical Cr concentration is exceeded, in which there is no structural relaxation [68].

The ranking of MO_2_ oxides in the scales of the alloys OHS1 and ZF6 in decreasing order of thermal conductivity (κ) and Young’s modulus (E) is SiO_2_, GeO_2_, TiO_2_, and SnO_2_, and the ranking in increasing order of linear coefficient of thermal expansion (α) is SiO_2_, TiO_2_, GeO_2_, and SnO_2_ [69] (the SnO_2_ oxide was not confirmed by GXRD, but the microanalysis suggested the presence of Sn in the scale; see Section 3.3 where the SnO_2_ has the same crystal structure as TiO_2_). A rapid temperature decrease is accompanied by temperature and stress gradients that could cause cracking and damage. The properties α, E, and κ are crucial for the response of oxides to rapid changes in temperature, which are described by the intrinsic thermal shock parameter (units W∙m^−1^∙MPa^−1^). The ranking of MO_2_ oxides and alumina in terms of decreasing intrinsic thermal shock parameter is SiO_2_, Al_2_O_3_, TiO_2_, GeO_2_, and SnO_2_ [69]. The scale of ZF6 contained MO_2_ oxides at the extremes of the aforementioned ranking for thermal shock, namely, SiO_2_ and GeO_2_, compared with the scale of OHS1 where the presence of Al_2_O_3_, TiO_2_, and SnO_2_ may have contributed to a more “measured” response to stresses.

Another possible source of reduced stress could be the presence of the Ti_2_Nb_10_O_29_ oxide in the scale of OHS1. This oxide was not found in the scale of ZF6, and the TiNb_2_O_7_ and AlNbO_4_ oxides were present in the scales of both alloys. The presence of Ti_2_Nb_10_O_29_ and TiNb_2_O_7_ in the scale that formed on Nb-39Ti (at.%) (Nb-25 wt.% Ti) after extended (≥100 h) oxidation between 750 and 780 °C was reported by Felten [70]. The CTE values of oxides in scales of Nb-silicide-based alloys were given in Reference [71]. The Ti_2_Nb_10_O_29_ has a negative CTE (−3.1 × 10^−7^/°C) compared with the CTE values of TiNb_2_O_7_ (4.5 × 10^−7^/°C) and AlNbO_4_ (39.7 × 10^−7^/°C) [72]. Furthermore, Nb_2_O_5_ and mixed (Nb_2_O_5_)_1−x_(TiO_2_)_x_ oxides (x = 0.05, 0.08, 0.11) that have negative CTE below 500 °C [73] could form owing to stoichiometric changes in oxides in the scale (see above). Even though GXRD did not provide evidence for the latter mixed oxides, it is suggested that the presence in the scale of OHS1 of oxide(s) with negative CTE decreased thermal expansion-related stresses in the scale and improved its adherence to the substrate.

In the Nb_2_O_5_–GeO_2_ system, partial melting was observed for 85 mol.% Nb_2_O_5_–15 mol.% GeO_2_ above 1090 ± 10 °C [74]. This would suggest that some liquation might have occurred in the scale of ZF6 that contained both these oxides, which were not present in the scale of OHS1. Thus, it is proposed that the simultaneous addition of Ge and Sn in the alloy OHS1 resulted in non-spallation of the scale that formed at 1200 °C because (a) the scale was able (i) to relax structurally owing to it containing AlNbO_4_ with low Cr concentration, and (ii) to respond in a “measured” way to stresses arising for temperature gradients owing to it containing SiO_2_, Al_2_O_3_, TiO_2_ and SnO_2_, and Ti_2_Nb_10_O_29_ with TiNb_2_O_7_, and AlNbO_4_, and (b) there was no liquation in the scale.

### 4.4. Alloy OHS1: High-Entropy Alloy (HEA) or Complex Concentrated Alloy (CCA)?

The concentrations of elements in a multicomponent high-entropy alloy (HEA) should be between 35 and 5 at.%. Some alloys that were studied recently do not adhere to this “definition” because they have an element with a concentration that exceeds 35 at.%, and other element(s) with concentration less than 5 at.%; for example, “HEAs” Fe_(64−x)_Mn_x_Ni_27.7__±1.3_Co_5.6__±0.3_Cr_2.3__±0.1_ (x = 21, 24, 27) were studied in Reference [75]. Such alloys “satisfy the general intent to explore complex phase space” [76], and they are currently also referred to as HEAs [75] or as complex concentrated alloys (CCAs) [76].

The alloy OHS1 does not meet the currently accepted definition of HEA, but it is a CCA with ΔH_mix_ = −41.24 kJ∙mol^−1^, ΔS_mix_ = 10.06 J·mol^−1^·K^−1^, VEC = 4.445, δ = 9.25, Δχ = 0.166, and Ω = 0.51 (parameters calculated for the actual composition of the cast alloy, see Section 3.1). The parameters VEC, δ, and Δχ are in the ranges of the parameters of a bcc solid solution and intermetallic HEAs [77]. For OHS1 the alloy design methodology NICE [10] predicts average weight gains of 3.5 and 36 mg/cm^2^, respectively, for 800 and 1200 °C, and creep rate 1.25 × 10^−5^ s^−1^ at 1200 °C and 170 MPa. The predicted weight gains are in good agreement with the measured ones (Table 3), and the creep rate does not meet the creep goal for Nb-silicide-based alloys [2,10], which is not surprising when the above parameters of OHS1 and the ratio *sd*/*sp* (= 2.1) are compared with the data in Figures 6 and 7 in Reference [77]. OHS1 could be a good “basis alloy” for alloy design to develop Nb-silicide-based alloys with a balance of oxidation and creep properties.

**Figure 15:**
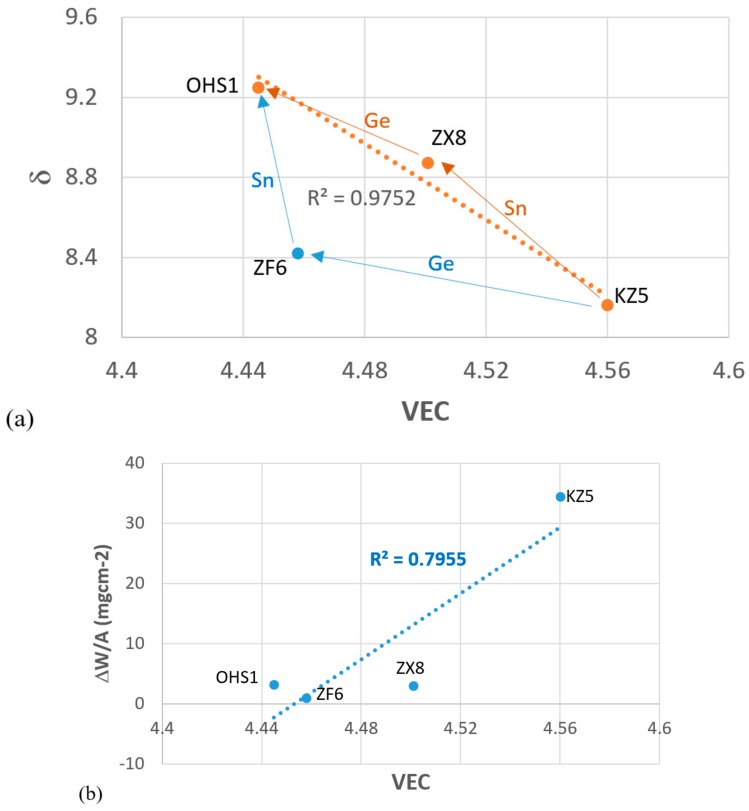
Plots (**a**) of the parameters δ and VEC and (**b**) of weight change per unit area at 800 °C versus VEC.

Figure 15a shows a map of the parameters δ (related to atomic size) and VEC (number of valence electrons per atom filled into the valence band) for the alloys OHS1, ZX8, ZF6, and KZ5. These parameters, together with the parameter Δχ (related to electronegativity), describe the alloying behavior of Nb-silicide-based alloys [77], and they were calculated using the actual alloy compositions [33,34,44]. Figure 15b shows the weight change per unit area (ΔW/A) of the alloy OHS1 at 800 °C versus its parameter VEC. According to the alloy design methodology NICE [10], (a) the trends of VEC and δ should be opposite for oxidation resistance, and (b) alloy design should aim to decrease the former and increase the latter. Indeed, this is the case for the parameters VEC and δ (Figure 15).

In Figure 15a, with KZ5 as the “basis” (reference) alloy, the blue arrows show changes of parameters with the addition of Ge and (Ge and) Sn in the alloys ZF6 and OHS1, the orange arrows show changes of parameters with the addition of Sn and (Sn and) Ge in the alloys ZX8 and OHS1, and the *R*^2^ value corresponds to the linear fit of the data for the alloys KZ5, ZX8, and OHS1, which is very good (*R*^2^ > 0.975) and better than that between the alloys KZ5, ZF6, and OHS1. Figure 15a shows “a direction of travel” for future alloy design for balance of properties that can be pursued within the framework of the alloy design methodology NICE [10].

Figure 15a shows (i) that the additions of Ge or Sn individually or simultaneously resulted in a decrease or increase, respectively, of the parameters VEC and δ, in agreement with NICE [10], (ii) that the addition of Ge in ZF6 resulted in a significant decrease in VEC with a small increase in δ, and (iii) that the addition of Sn in OHS1 resulted in a small decrease in VEC and a significant increase in δ. Figure 15b shows that the additions of Ge or Sn individually or simultaneously resulted in lower VEC values and lower weight changes compared with the alloy KZ5, in agreement with NICE [10].

## 5. Summary and Concluding Remarks

This research sought to understand how the simultaneous addition of Ge and Sn in Nb-silicide-based alloys affects their oxidation resistance. We presented the results for the Hf-free Nb-24Ti-18Si-5Al-5Cr-5Ge-5Sn alloy that was selected to provide answers to the questions that were asked in the introduction of this paper. The alloy exhibited macrosegregation of Si which was reduced owing to the addition of both Ge and Sn. The Nb_ss_ was present in the cast alloy at a low volume fraction and was not stable after the heat treatment at 1400 °C. The A15-Nb_3_Sn and C14-NbCr_2_ Laves were stable phases. The primary βNb_5_Si_3_ did not transform to αNb_5_Si_3_ after the heat treatment. The additions of Ge and Sn suppressed pest oxidation at 800 °C. There was no spallation of the scale that formed at 1200 °C. There was enrichment in Ge and Sn in the substrate below the scale/substrate interface where the compounds Nb_3_Sn, Nb_5_Sn_2_Si, (Ti,Nb)_6_Sn_5_, and Nb_5_Ge_3_ were formed. In the bulk of the alloy, after the oxidation at 1200 °C, the solid solution was very rich in Ti. Improvement of oxidation resistance at both temperatures was accompanied by a decrease and increase, respectively, of the alloy parameters VEC and δ, in agreement with the alloy design methodology NICE.

The interesting finding of this piece of experimental work was the elimination of scale spallation at 1200 °C in the alloy OHS1. This was attributed (a) to the formation of a Ti-rich (Ti,Nb)_ss_ solid solution and (Ti,Nb)_6_Sn_5_, respectively, in the bulk and below the scale, (b) to the low concentration of Cr in the scale, (c) to the absence of GeO_2_ in the scale, (d) to the formation of αAl_2_O_3_ in the scale, and (e) to the presence (i) of Nb_5_Ge_3_ below the scale/substrate interface and (ii) of oxides in the scale, namely, SiO_2_, Al_2_O_3_, TiO_2_, and SnO_2_, and Ti_2_Nb_10_O_29_,TiNb_2_O_7_, and AlNbO_4_, respectively, with a range of intrinsic thermal shock resistances and CTE values that reduced stresses in the scale and the substrate below it.

## Figures and Tables

**Table 1:** Microanalysis data (at.%) of OHS1-AC.

Area/Phase	Nb (at.%)	Ti (at.%)	Si (at.%)	Cr (at.%)	Al (at.%)	Ge (at.%)	Sn (at.%)
Top	40.0 ± 1.1	23.2 ± 1.4	18.8 ± 1.6	4.3 ± 0.6	4.6 ± 0.4	5.2 ± 0.4	3.9 ± 0.5
	42.5–38.5	25.0–20.4	22.3–15.9	5.1–3.3	5.3–3.9	5.6–4.5	4.8–3.1
Center	41.6 ± 1.7	22.1 ± 1.7	18.7 ± 1.6	4.1 ± 0.5	4.7 ± 0.5	4.7 ± 0.5	4.1 ± 0.4
	43.6–38.0	25.4–20.1	21.2–15.5	5.3–3.2	5.6–3.4	5.7–3.7	5.1–3.2
Bottom	39.2 ± 0.6	24.2 ± 0.8	17.9 ± 0.8	4.8 ± 0.4	4.8 ± 0.4	5.2 ± 0.3	3.9 ± 0.4
	40.7–38.0	25.8–22.8	19.1–16.7	5.4–4.2	5.6–4.0	5.6–4.7	4.5–3.4
Nb_ss_	23.3 ± 0.7	30.6 ± 1.0	5.3 ± 1.0	29.4 ± 1.6	7.2 ± 0.7	1.5 ± 0.5	2.7 ± 0.2
	24.3–22.2	32.1–29.5	6.8–3.9	31.9–27.4	8.4–6.3	2.2–0.7	3.1–2.4
NbCr_2_	23.1 ± 1.1	17.6 ± 1.2	7.3 ± 0.4	45.3 ± 2.6	5.1 ± 0.4	0.9 ± 0.1	0.7 ± 0.4
	24.9–21.7	19.6–16.2	8.0–7.0	48.2–42.0	5.8–4.5	1.1–0.9	1.4–0.3
Nb_3_Sn	49.0 ± 0.9	24.9 ± 0.5	2.9 ± 0.1	4.4 ± 0.3	5.8 ± 0.3	1.6 ± 0.2	11.4 ± 0.4
	50.3–47.8	25.7–24.3	3.1–2.7	4.6–3.8	6.2–5.3	1.9–1.4	12.1–11.0
Ti-rich Nb_3_Sn	41.9 ± 0.9	29.4 ± 1.0	2.8 ± 0.2	7.4 ± 0.7	7.3 ± 0.3	1.5 ± 0.1	9.7 ± 0.5
	43.2–40.4	31.2–27.7	3.2–2.4	8.2–6.4	7.8–6.8	1.7–1.3	10.5–8.8
Nb_5_Si_3_	43.6 ± 0.3	18.6 ± 0.3	27.3 ± 0.5	1.2 ± 0.1	2.0 ± 0.2	6.2 ± 0.1	1.1 ± 0.2
	43.8–43.1	19.1–18.5	27.7–26.7	1.3–1.1	2.2–1.8	6.3–6.1	1.4–1.0
Ti-rich Nb_5_Si_3_	38.8 ± 2.0	23.0 ± 1.9	20.6 ± 0.6	1.9 ± 0.4	5.2 ± 0.4	6.7 ± 0.2	3.8 ± 0.2
	40.5–36.1	25.3–21.1	21.3–19.6	2.5–1.6	5.8–4.8	7.1–6.4	4.1–3.5

**Table 2:** Microanalysis data (at.%) of OHS1-HT.

Area/Phase	Nb (at.%)	Ti (at.%)	Si (at.%)	Cr (at.%)	Al (at.%)	Ge (at.%)	Sn (at.%)
Top	39.6 ± 0.4	24.1 ± 0.5	18.2 ± 0.8	4.2 ± 0.3	4.6 ± 0.3	5.1 ± 0.2	4.2 ± 0.3
	39.9–39.1	24.6–23.6	19.2–17.4	4.5–3.9	5.0–4.2	5.3–4.8	4.7–4.0
Bulk	40 ± 1.0	23.7 ± 0.6	17.8 ± 1.0	4.6 ± 0.4	4.7 ± 0.2	5.0 ± 0.2	4.2 ± 0.5
	41.1–38.4	24.6–23.2	19.1–16.3	5.0–4.0	5.0–4.5	5.3–4.6	5.2–3.8
Bottom	39.4 ± 0.6	24.4 ± 0.4	18.0 ± 0.7	4.4 ± 0.5	4.5 ± 0.2	5.2 ± 0.2	4.1 ± 0.2
	40.3–38.8	24.9–24.0	18.6–17.1	4.9–3.8	4.7–4.3	5.4–5.0	4.3–3.8
NbCr_2_	26.0 ± 0.6	10.1 ± 0.7	8.8 ± 0.2	50.5 ± 1.0	3.5 ± 0.4	0.7 ± 0.0	0.4 ± 0.3
	26.6–25.3	10.5–9.1	9.1–8.6	51.7–49.3	3.9–3.1	0.8–0.7	0.7–0.1
Nb_3_Sn	47 ± 0.4	23.8 ± 0.4	2.6 ± 0.2	5.8 ± 0.2	5.0 ± 0.6	1.3 ± 0.1	14.5 ± 0.8
	47.5–46.7	24.1–23.1	2.8–2.4	6.1–5.5	5.5–4.0	1.3–1.2	15.8–14.0
Nb_5_Si_3_	37.1 ± 0.4	24.2 ± 0.8	22.0 ± 1.3	3.2 ± 0.3	4.8 ± 0.3	5.8 ± 0.7	2.9 ± 0.4
	37.6–6.5	25.0–23.5	23.5–20.9	3.5–3.0	5.0–4.5	6.4–5.0	3.2–2.5
Ti-rich Nb_5_Si_3_	33.2 ± 0.2	27.2 ± 1.3	24.9 ± 0.7	2.1 ± 0.4	4 ± 0.5	4.9 ± 0.7	3.7 ± 0.2
	34.8–32.6	29.5–26.6	25.6–24.1	2.7–1.7	4.9–3.7	6.7–5.1	4.1–3.5

**Table 3:** Total weight gain and oxidation rate constants of OHS1 at 800 and 1200 °C.

Temperature	n	K_l_ (g·cm^−2^·s^−1^)	K_p_ (g^2^·cm^−4^·s^−1^)	Weight Gain (mg/cm^2^)
800 °C	0.46	-	2.4 × 10^−11^ (0–100) h	3.19
1200 °C	0.83	1.1 × 10^−7^ (> 3.1) h	4.9 × 10^−10^ (0–3.1) h	31.28

**Table 4:** Alloy parameters for the macrosegregation of Si (MACSi) in the cast alloys KZ5, ZF6, OHS1, and ZX8.

Alloy	ΔH_m_ (kJ/mol)	T_m_ (K)	ΔH_m_/T (J/mol·K)	ΔH_m_^sd^/ΔH_m_^sp^	T_m_^sd^ (K)	T_m_^sp^ (K)	T_m_^sd^/T_m_^sp^	[ΔH_m_/T_m_] × [ΔH_m_^sd^/ΔH_m_^sp^]^−1^	MACSi (at.%)
ZX8 OHS1 ZF6 KZ5	26.9 27.7 27.7 27.5	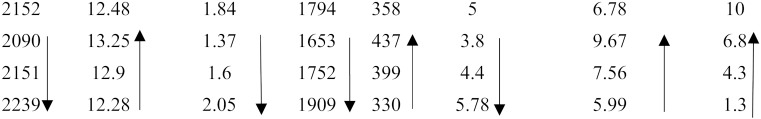							

**Table 5:** Alloy parameters for the macrosegregation of Si in the cast alloys KZ7, ZF5, ZX6, and OHS1 considering the Al effect with Ge or Sn individually and simultaneously. Bold red numbers and arrows represent strong trends, while non-bold red numbers and arrows represent weak trends.

Alloy	ΔH_m_ (kJ/mol)	T_m_ (K)	ΔH_m_/T_m_ (J/mol·K)	ΔH_m_^sd^/ΔH_m_^sp^	T_m_^sd^ (K)	T_m_^sp^ (K)	T_m_^sd^/T_m_^sp^	[ΔH_m_/T_m_] × [ΔH_m_^sd^/ΔH_m_^sp^]^−1^	MACSi (at%)
OHS1 ZX6 (Al) ZF5 (Al) KZ7 (Al)	27.7 27.3 28 27.7	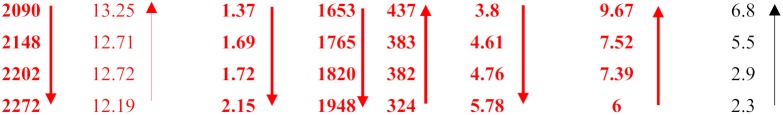							

**Table 6:** Alloy parameters for the macrosegregation of Si in the cast alloys KZ4, ZF4, ZX4, and OHS1 considering the Cr effect with Ge and Sn individually (indicated by (Cr) next to the alloy designation) and simultaneously (alloy OHS1). Bold red numbers and arrows represent strong trends (excluding the Sn + Ge synergy in OHS1), while bold italics represent minima (in purple) and maxima (in green).

Alloy	ΔH_m_ (kJ/mol)	T_m_ (K)	ΔH_m_/T_m_ (J/mol·K)	ΔH_m_^sd^/ΔH_m_^sp^	T_m_^sd^ (K)	T_m_^sp^ (K)	T_m_^sd^/T_m_^sp^	[ΔH_m_/T_m_] × [ΔH_m_^sd^/ΔH_m_^sp^]^−1^	MACSi (at%)
ZX4 (Cr) OHS1 ZF4 (Cr) KZ4 (Cr)	27.8 27.7 28.4 28.2